# From random to predictive: a context-specific interaction framework improves selection of drug protein–protein interactions for unknown drug pathways

**DOI:** 10.1093/intbio/zyac002

**Published:** 2022-03-21

**Authors:** Jennifer L. Wilson, Alessio Gravina, Kevin Grimes

**Affiliations:** 1 Department of Bioengineering, University of California Los Angeles, Los Angeles, CA, USA; 2 Department of Computer Science, University of Pisa, Pisa, Italy; 3 Department of Chemical and Systems Biology, Stanford University, Stanford, CA, USA

**Keywords:** drug pathways, protein network modeling, side effect prediction

## Abstract

With high drug attrition, protein–protein interaction (PPI) network models are attractive as efficient methods for predicting drug outcomes by analyzing proteins downstream of drug targets. Unfortunately, these methods tend to overpredict associations and they have low precision and prediction performance; performance is often no better than random (AUROC ∼0.5). Typically, PPI models identify ranked phenotypes associated with downstream proteins, yet methods differ in prioritization of downstream proteins. Most methods apply global approaches for assessing all phenotypes. We hypothesized that a per-phenotype analysis could improve prediction performance. We compared two global approaches—statistical and distance-based—and our novel per-phenotype approach, ‘context-specific interaction’ (CSI) analysis, on severe side effect prediction. We used a novel dataset of adverse events (or designated medical events, DMEs) and discovered that CSI had a 50% improvement over global approaches (AUROC 0.77 compared to 0.51), and a 76–95% improvement in average precision (0.499 compared to 0.284, 0.256). Our results provide a quantitative rationale for considering downstream proteins on a per-phenotype basis when using PPI network methods to predict drug phenotypes.

## INTRODUCTION

Analyzing protein pathways downstream of drug targets is an attractive approach for understanding drug effects; however, these methods are not currently used to prioritize drug targets because of their tendency to overpredict drug phenotype associations. There’s ample motivation for understanding drug effects on cellular pathways and using protein interactions to anticipate resulting phenotypes [1–3]. Pathways analyses have been useful in understanding disease mechanisms [4, 5] by linking disease-associated genes through protein–protein interactions. Similarly, protein–protein interactions (PPIs) have also linked low-signal genetic associations from genome-wide association studies [6, 7]. Linking drug targets to disease-associated genes via PPIs has also been used for identifying repurposing opportunities [8–12] and for predicting drug combination effects [13, 14]. Interestingly, linking drug targets to phenotype-associated genes predicts many more drug–phenotype relationships than are supported by the evidence. These additional predictions are often considered to be false positives because we lack gold standards of negative, or no-effect, relationships between drugs and phenotypes [15]. This has limited the prediction performance and precision of using PPIs to anticipate drug effects. A recent approach, which leveraged PPIs for understanding drug mechanisms, reported precision values of 0.064–0.091 for theirs and comparator approaches [16].

Multiple pathway–based searches have leveraged disease-specific information to prioritize drug targets; however, these successes were limited to single disease applications. From these applications, we hypothesized that phenotype-specific information could improve predictive performance of PPI approaches. Specific pathway examples span many disease areas including epilepsy [17], Huntington’s disease [18], clear cell renal cell carcinoma [19] and breast cancer [20], but each example explores targets for a single disease. In comparison, existing PPI approaches have used global methods where similar rules—distance thresholds or statistical thresholds—are applied to simultaneously scrutinize several drug–phenotype relationships, both for drug safety and efficacy. While we hypothesized that incorporating phenotype-specific information into PPI methods could improve performance, we lacked quantitative evidence for this approach.

We aimed to understand the effect of incorporating phenotype-specific information on PPI predictive performance and discovered a striking increase in performance and precision by incorporating this information. Because we lacked drug null effect data, we pursued an innovative strategy using drug–side effect relationships extracted from drug labels. We narrowed our data to the most severe side effects (e.g. myocardial infarction, pancreatitis), referred to as designated medical events (DMEs), from the warnings, boxed warnings, and precautions sections of the drugs’ labels because this subset of side effects has received consistent FDA review, compared to ‘milder’ adverse events (e.g. nausea, rash). We reasoned that drugs without a DME listed on the drugs’ label are likely not causative for the side effect or caused the side effect in rare circumstances. Further, we assumed that labeled warnings for severe side effects were less sensitive to underreporting or heterogeneity in reporting rates observed for all side effects [21]. Using this assumption, we generated a novel and useful dataset of positives (drugs with the side effect on their label) and negatives (drugs without the side effect on their label). Additionally, unintended drug side effects are a major contributor to drug attrition [22, 23] and improving side effect prediction performance could be of great utility to the PPI and drug development communities. We used interpretable machine learning to analyze labeled and unlabeled drugs with a PPI association to a side effect DME and measured the impact of this data on prediction performance and precision.

## RESULTS

### Statistical thresholding cannot clearly separate true and false positives

**Figure 1.**
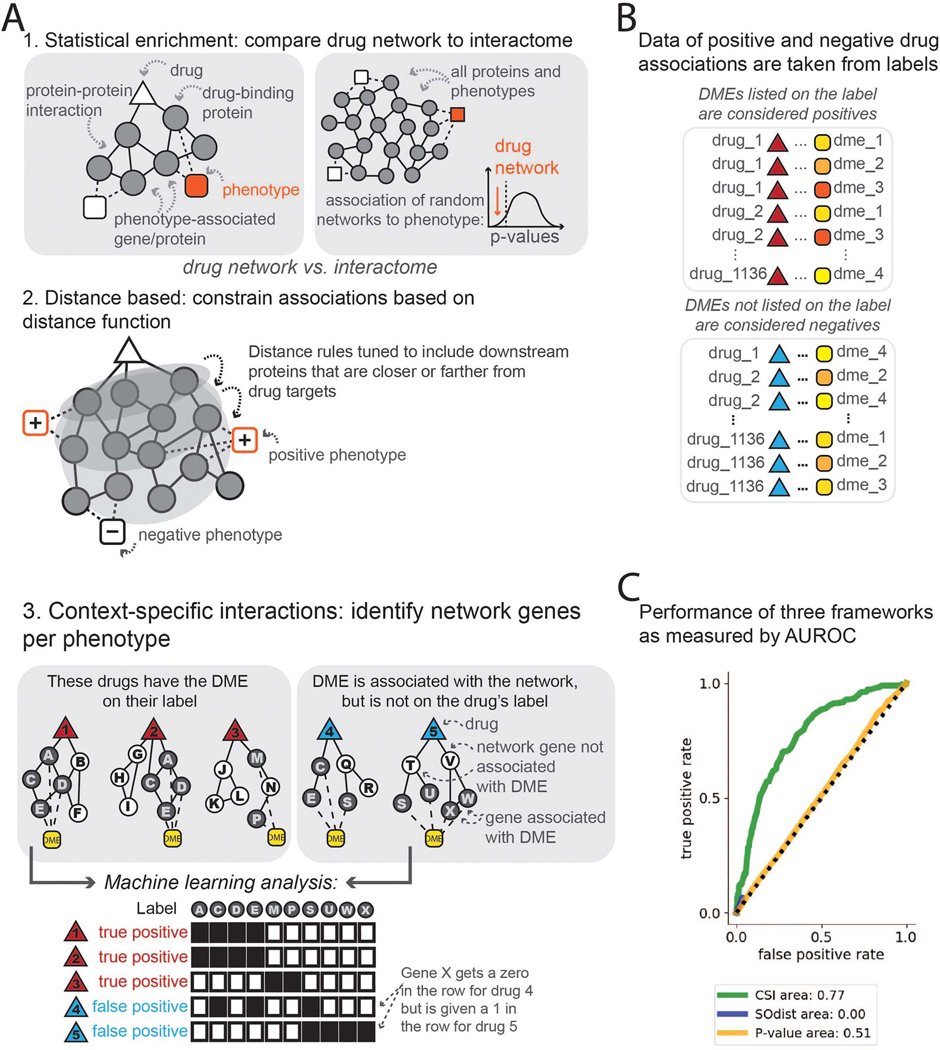
Consideration of three frameworks shows superior performance of context-specific analysis. (A) We considered three frameworks: 1. statistical enrichment—a network association is selected if the drug’s PPI network is enriched for associations to a phenotype of interest relative to random, input-matched networks. 2. distance-based—an interaction distance function is calibrated to identify positive phenotypes and minimizing false positive phenotypes. 3. context-specific interactions (CSI) analysis—machine learning analysis (e.g. logistic regression) is used to discover which downstream genes/proteins and interactions separate true from false positives. (B) Positive drug–DME relationships are extracted from the warnings, boxed warnings, and precautions section of the drug’s label. Negative cases (or cases where the drug is not expected to cause the DME) are inferred from the absence of the DME on the drug’s label. Red or blue triangles represent positive or negative drugs, and multiple shades of yellow/orange are meant to distinguish different DMEs in the dataset. (C) ROC curves for distinguishing true and false positives using *P*-value (orange) or a distance-based approach (blue) or using CSIs (green). Legend indicates AUROC value for each framework.

We first investigated a statistical threshold method (Fig. 1A) for separating true positives and true negatives. The motivation for this approach is that setting a significance threshold is relatively easy to implement, and theoretically, true positive phenotypes may be more significantly associated with drug networks than true negative phenotypes. In our case, we specifically explored whether DMEs from the drug’s labels were more significantly associated with a drug’s network than DMEs not listed on the drug’s label. For this analysis, we used PathFX [9] in its original published form and altered the significance threshold for filtering network-associated phenotypes. Briefly, PathFX used a network propagation technique, starting from a drug’s target proteins, to identify a network of relevant protein–protein interactions from a larger interactome network (further described in Materials and Methods, Supplemental Fig. 1). The algorithm used a database of gene–phenotype associations and statistical enrichment to identify enriched network phenotypes relative to the original interactome. Of the 1970 drugs, 997 drugs had drug target proteins from DrugBank [24] and at least one interaction partner in our interactome. PathFX required drug target proteins to seed the network propagation search. We used PathFX to create networks for these 997 drugs and investigated where PathFX identified a true positive—a network association between a drug and a DME on the drug’s label—and a false positive— a network association to a DME not listed on the drug label, at increasing score thresholds. We investigated the multiple hypothesis–corrected *P*-values and the normalized *P*-values. The normalized *P*-values represent the ratio of the multiple hypothesis–corrected *P*-value for a phenotype to the median of all multiple hypothesis– corrected *P*-values derived from 100 input-matched random networks. PathFX used 100 random networks each generated with input-matched drug-binding proteins to measure the distribution of drug–phenotype associations and calculated this distribution per phenotype (to account for the fact that phenotypes are associated with a wide range of total proteins). The distributions for these *P*-values, both raw and normalized, overlap (Supplemental Fig. 2), and when we plotted sensitivity and specificity on the ROC graph, the area under the receiver operator curve (AUROC) is 0.51 (Fig. 1C). These results suggested that a global statistical threshold for predicting drug–phenotype relationships is insufficient for separating true and false positives.

### Using a distance-based approach does not increase prediction performance

We next investigated a distance metric for separating true and false positives (Fig. 1A). Conceptually, we were motivated to understand if including PPIs that were ‘close’ to or ‘far’ from drug target proteins improved our ability to discern true from false positives. The assumption in this framework is that drug effects are only propagated to proteins within a defined proximity relative to the drug target proteins. In this paradigm, ‘close’ signaling molecules might interact directly with a drug target, and ‘far’ signaling molecules may be several protein–protein interactions removed from a drug’s target. As mentioned, many approaches have used network propagation and most network propagation methods tune the ‘distance’ before pursuing classification or prediction. To measure performance at ‘close’ and ‘far’ distances, we modified PathFX from the original published form (Supplemental Fig. 1). Specifically, the original PathFX algorithm relied on an empirically derived path score threshold to minimize common biases for network algorithms including hub bias (a gene/protein has high connectivity because it is well studied; this is further explained in methods). Although other network propagation techniques have not used the same empirical path score, we considered this path score to be a sufficient proxy for understanding how tuning interaction path distance can affect prediction performance. To measure the effect of tuning interaction path distance, we created modified versions of PathFX using nonoptimal path score thresholds (e.g. PathFX_dist0.9, PathFX_dist0.8, etc., further explained in Materials and Methods). Conceptually, a higher or lower score threshold forced PathFX to analyze proteins ‘closer’ or ‘farther’ from drug targets, respectively. In our interaction network, edges are scored from 0 to 1.0; edge scores reflected the likelihood of an interaction derived from a corpus of data and high-quality interactions had higher scores (further described in Materials and Methods). When modifying PathFX, a path score of 0.99 was sufficiently high enough to exclude any downstream network proteins and ‘networks’ at this score effectively only included drug targets. We reanalyzed the 997 drugs mentioned above using these distance-constrained versions and investigated how including ‘farther’ downstream proteins affected performance. We had anticipated that performance would increase initially and then decline as we included downstream proteins that were too far from the targets. At distances of 0.82–0.99, we were unable to generate a full ROC curve (Fig. 1C) because the sensitivity and specificity did not improve at increasing distances. We discovered that modifying the path score threshold did not increase an ability to detect true positive associations to DME-associated genes. This suggests that global propagation away from drug target(s) may not be sufficient for discerning true from false positive phenotypic associations.

### Context-specific interactions increase ability to discern true from false positive DME associations

Much of biology is context dependent meaning that the molecular drivers of a pathway are specific to that pathway context. Indeed, many pathway investigations have used disease-specific pathways to uncover target candidates for therapeutic interventions [17, 18, 20]. We hypothesized that each DME may result from association to DME-specific downstream proteins and that a better separator of true and false positives could be the specific network genes/proteins supporting an association to a DME phenotype. The assumption in this context-specific interaction (CSI) framework is that drug phenotypes result from association to specific proteins. For example, we hypothesized that of all drug networks associated to myocardial infarction genes (or any other DME), that drugs with the labeled warning (true positives) would share network proteins that were distinct from drug networks where the DME was not on the label (false positives). To test this hypothesis, we combined PathFX, a network propagation technique, with subsequent machine learning analysis. We generated matrix files for all drugs associated with a DME where each row represented a drug, and each column represented a DME-associated gene in a drug’s network (Fig. 1A3). Each row was labeled as a true positive (the DME was on the drug’s label) or a false positive (the DME was not on the drug’s label but was associated with the drug’s network). We tested logistic regression, decision trees, and random forest algorithms for their ability to distinguish true from false positives on a per-DME–phenotype basis. We performed nested cross-validation to select among these methods and used the F1 statistic to discover that these methods were comparable in performance (Fig. 1A, Supplemental Fig. 3, Supplemental Table 1). We selected the F1 statistic because the number of true and false positives was not balanced, and this metric is more stable when there is class imbalance. We selected a simple logistic regression because it was the most straightforward method for interpretation and next generated a logistic regression model per DME. However, we were only able to conduct cross-validation for DMEs where we had at least 10 true and false positive examples and this reduced our analysis to 16 DMEs.

Using a logistic regression model combined with networks discovered for DME-associated drugs increased AUROC values 50% over statistical thresholds (AUROC 0.77 compared to 0.51) or distance methods (Fig. 1C) when investigating all DMEs. Performance varied for each DME (Supplemental Fig. 4). To understand differences in performance per DME, we investigated multiple performance metrics—F-score, precision, recall and AUROC— and their relationship to features of the input data—total number of true positives, total number of false positives, ratio of true positives to false positives, fraction of all cases that are true positives, total number of genes associated to the DME, number of genes appearing in only a single network and the fraction of genes that are shared between true and false positive drug networks and the correlation of performance metrics to these data features (Supplemental Fig. 5, Supplemental Table 2). The average *F*-score and average precision were moderately correlated (*R* ∼ 0.6) with the ratio of true to false positives used for model training and were moderately negatively correlated (*R* ∼ −0.6) with the number of false positives (Supplemental Fig. 5B). This suggested that context-specific modeling efforts are improved when balanced datasets of true and false positives are available. Model performance was not affected by the total number of genes, or the proportion of genes shared between true and false positive networks. The latter results highlighted that classification depends on the combinatorial effect of genes and further emphasized the need to simultaneously study multiple proteins downstream of drug effects instead of just single targets. We further investigated precision of each method (Table 1) because many network methods have suffered from low precision. We discovered that a CSI approach improved precision 76% (0.499 vs 0.284) compared to the statistical threshold approach and 95% (0.499 vs 0.256) compared to the distance approach (Table 1).

**Figure 2.**
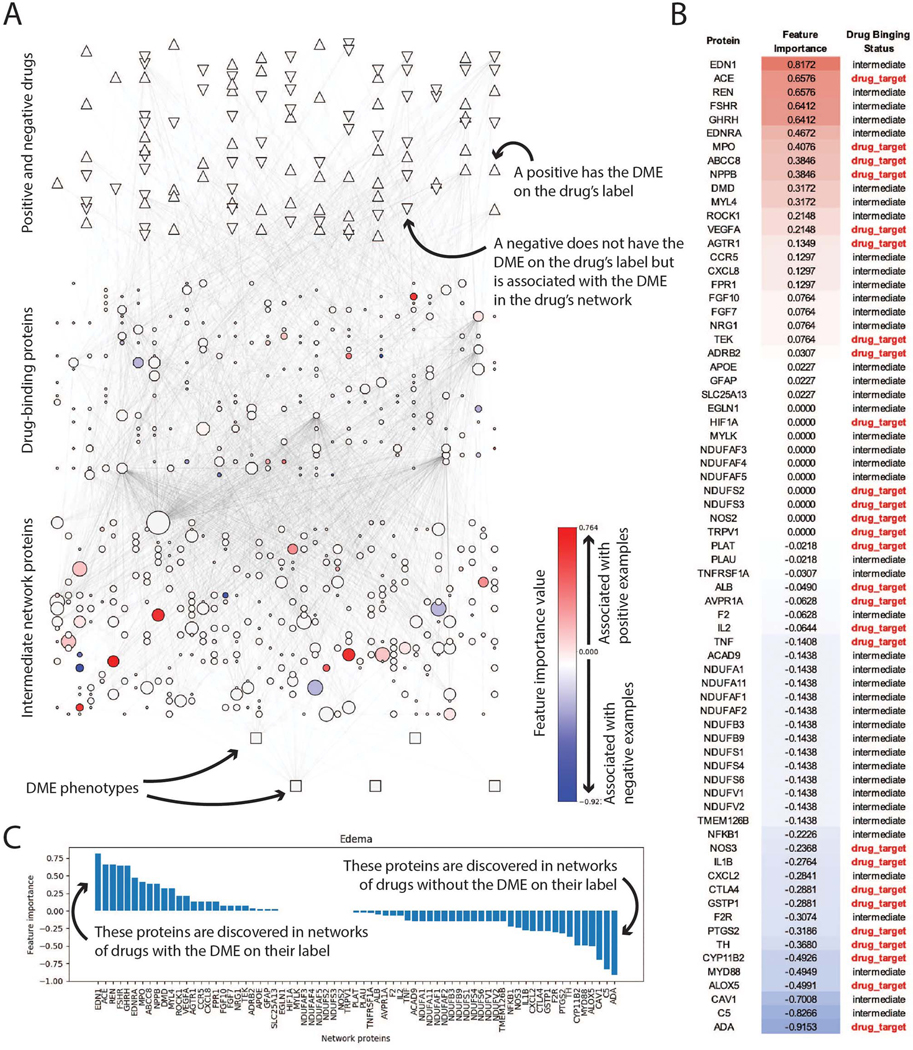
Meta-analysis of DME-associated merged network identified CSIs for edema. The merged interaction network for all true and false positive drugs associated with edema highlighted which network components—drug-binding and network proteins—have high feature importance in the logistic regression model (A). True/false positive drugs are represented in the top layer as upright/inverted triangles, respectively. Drug-binding and intermediate pathway proteins are represented in the second and third layers. The size of the protein reflects the number of networks in which the protein appears. Relevant edema-associated phenotypes are represented as boxes in the last layer. Protein coloring reflects the feature importance in the logistic regression model. Red/blue coloring represents association to true/false positive networks. We have also provided tabular results (B) indicating protein feature importance score and whether the protein is drug-binding and a histogram (C) of ranked feature importance scores.

CSIs are further attractive for their interpretability. For instance, logistic regression feature importance scores highlighted network proteins—both drug targets and downstream proteins—that are associated with true and false positive drugs for each DME. We investigated the feature importance scores generated by logistic regression for all DMEs and highlighted an example for edema in Fig. 2. For all other DMEs, merged network images are included in Supplemental File 1 (Supplemental Figs 6–20) and feature importance scores are contained in Supplemental File 2. We overlaid feature importance scores on a merged network for edema to visualize these scores in the context of drug protein–protein interaction networks (Fig. 2). The merged network contains the union of all protein–protein interactions for true and false positive drug networks associated with edema. Interestingly, the logistic regression prioritized more downstream than target proteins as indicated by more red/blue protein shading in the lower-middle layer of the merged PPI network and by the protein type in the figure table. Further, higher feature importance scores could be used to prioritize and test downstream proteins for their role in modulating drug effects. For instance, endothelin 1 (EDN1) had the highest feature importance score in the model, and it was an ‘intermediate’ or downstream protein (none of the edema-associated drugs targeted this protein) discovered in multiple drug networks. Administration of endothelin-1 to mice has been shown to increase edema [25]. Thinking mechanistically, this could suggest that drug-induced edema is caused by increased EDN1 protein that is stimulated downstream from drug-targeted proteins. The endothelin receptor type A (EDNRA) also has high feature importance and is an intermediate network gene, further suggesting that the endothelin pathway may modulate druginduced edema. Our analysis hypothesizes that drug targets proximal to the endothelin pathway are more likely to have effects on edema, which may be a drug development risk. Follicle-stimulating hormone receptor (FSHR) was also a downstream protein of many drugs associated with edema on their labels and FSHR had high feature importance. Literature supports that high doses of gonadotropins, including follicle-stimulating hormone, can result in edema [26]. Our method also prioritized vascular endothelial growth factor (VEGF), and anti-VEGF therapies are used for treating edema-related conditions such as diabetic macular edema, supporting the relevance of this protein for drug-induced edema. The cytokine, C-X-C Motif Chemokine Ligand 8 (CXCL8), more commonly known as interleukin 8 (IL8), is also associated with edema, but literature suggests a correlative relationship. Specifically, higher levels of IL8 are associated with diabetic macular edema [27] and inhibition of mechanistic target of rapamycin (MTOR) attenuated both IL8 release and lung edema in an LPS-induced model of lung injury [28]. While also associated with LPS-induced lung injury, our approach deprioritized C-X-C Motif Chemokine Ligand 2 (CXCL2) [29], suggesting that this protein is not associated with drug-induced edema. Additionally, reduced caveolin 1 (CAV1) expression levels were associated with greater brain edema in an influenza-associated encephalopathy model [30]; however, our model also deprioritized this scaffolding protein, suggesting that this molecule is also not associated with drug-induced edema. Experimental validation was outside the scope of this work, but these results highlight interesting opportunities for further validating these mechanistic predictions and the need to parameterize network relationships to understand the magnitude of downstream effects on drug-induced phenotypes. In this section, we illustrated the type of interpretation aided by our method for edema; however, the phenotype-specific models for other side effects could also be used to understand which downstream proteins could be modifying other drug-induced effects (feature importance scores for other DMEs are contained in Supplemental File 2).

## DISCUSSION

Protein–protein interaction networks are increasingly used to predict drug phenotypes; however, these methods tend to overpredict phenotype associations. We observed that many methods use global approaches for assessing all phenotypes and we hypothesized that developing phenotype-specific approaches would improve prediction performance. We compared two global approaches—statistical thresholding and distance thresholding—to our novel per-phenotype approach, ‘context-specific interactions’ (CSI). With CSI analysis, we measured a striking increase in model performance and precision when predicting adverse drug phenotypes. The CSI approach is additionally attractive as a ‘white-box’ machine learning approach. More specifically, the method prioritized protein–protein interactions downstream of drug targets that may represent a pathway for the drug-induced effects. We highlighted an example for edema where edema-causing drugs did not share the same targets, but they shared similar downstream proteins. We predicted downstream proteins for 15 other DMEs using this approach, suggesting novel insights for drug-induced side effects. Proteins downstream of positive drugs were distinct from proteins downstream of false positive drugs and literature evidence supports the possible relevance of these proteins. The major contribution of CSI analysis is that prediction performance and precision are improved by linking targets to downstream proteins associated with drug-induced phenotypes instead of using global methods for prioritizing downstream proteins.

Our CSI approach required examples of positive and negative drugs and was limited to prediction of drug side effects because we lacked sufficient negative cases for drug effects on disease. We made a useful assumption that drugs without a DME on its label were likely not causative of or rarely caused the side effect. However, there are many types of information that influence a labeled warning—including reporting rates and severity of the side effect—and thus, there may be limitations to this assumption. It would be important to test our predictions on a novel dataset or consider side effect severity when analyzing associated downstream proteins. Similarly, this limited our analysis to drug side effects and prevented analysis of drugs’ disease-modifying effects.

To expand CSI analysis for predicting a drug’s effect on disease, we would require several examples where drugs were tested and found to have no effect on a disease. We discovered that prediction performance and precision correlated with the number of positive and negative examples in the dataset; we observed that a relatively large number of false positives reduced model performance. Future CSI analyses will require more positive examples or computational techniques to improve class balance. Similar techniques have been used to extract adverse drug reactions from the electronic health records [21]. Because drugs aren’t routinely tested in multiple disease indications, a recent approach combined drug approvals with ongoing clinical studies to identify gold standard datasets for considering repurposing opportunities [31]. However, the scale of this dataset is still insufficient for considering the number of drug–phenotype associations discovered from PPI networks. Recent technological developments in observational studies using the electronic health record may provide an opportunity for developing sufficient examples. For instance, the LEGEND study simultaneously measured the effect of cardiovascular drugs on multiple cardiovascular outcomes [32]. Additionally, recent developments to the CohortMethod [33] package enabled simultaneous consideration of multiple ‘control’ outcomes for which a drug is expected to have no effect.

CSI ‘mining’ may advance PPI network predictions to have sufficient predictive power to inform decision-making at the level or regulatory review or industrial selection. Although curating positive and negative training examples is required for CSI analysis, we anticipate opportunities to use cell-based screens or phenotypic assays for deriving these examples. Already, we have explored using high-throughput screening and PPI networks as a means for mining CSIs relevant to psychiatric disease demonstrating the utility of experimental systems for deriving positive and negative examples. Further experimental validation would be needed to confirm the relevance of downstream proteins identified in CSI analysis. As an initial test of these predictions, we identified drug combinations where a drug interaction was predicted if the combo drug bound a DME-associated downstream protein. We further measured drug interaction effects on DMEs using the electronic health record (in preparation), further suggesting that CSIs identify downstream proteins relevant to drug-induced phenotypes. Together, these results suggest a paradigm shift toward network engineering of context-specific pathways to more effectively predict drug phenotypes.

## MATERIALS AND METHODS

### Extracting true positive and true negative drug examples from drug labels

We extracted relevant phenotypes from the drugs’ labels using a custom natural language processing (NLP) query (publication forthcoming, data included in Drugs_labeled_for_AEs.txt). In this query, we searched text in the ‘warnings’, ‘boxed warnings’, and ‘precautions’ portions of drugs’ labels and looked for specific designated medical event (DME) terms or synonyms. The selection of terms and synonyms were done in collaboration with FDA scientists using their in-house NLP pipeline for reading text and extracting relationships (publication forthcoming). We specifically searched for mentions of agranulocytosis, anaphylactic reaction, cardiac arrest, cerebral infarction, completed suicide, deep vein thrombosis, delirium, edema, gastric ulcer, generalized tonic–clonic seizure, hemolytic anemia, hemorrhage, hepatic necrosis, hyperlipidemia, hypertension, interstitial lung disease, myocardial infarction, myopathy, neuroleptic malignant syndrome, pancreatitis, peripheral neuropathy, pneumonia, proteinuria, pulmonary edema, QT prolongation, sepsis, serotonin syndrome, sleep apnea syndrome, sleep disorder, Stevens–Johnson syndrome, tardive dyskinesia, thrombocytopenia, ventricular tachycardia, and visual acuity reduced. This analysis yielded associations between 1970 drugs and 34 DMEs.

In the cases where a DME was listed on a drug’s label, we considered this a ‘positive’ drug. Interestingly, gold standard datasets for negative drug effects (e.g. a drug was tested for diabetes but does not affect the diabetes phenotype) are lacking [15]. Because FDA review of serious adverse reactions, including DMEs, is rigorous for all approved drugs, we reasoned that if a drug’s label did not list a DME, then the drug likely does not cause the DME or causes the DME rarely. This assumption provided a valuable opportunity for understanding network method performance when other gold standard datasets were lacking. Using this reasoning, we defined ‘negative’ drugs as any of the 1970 drugs in our drug set that do not have a DME listed on their drug label. We defined positives and negatives for each DME separately. For instance, 496 drugs were associated with myocardial infarction on their drug label, and these drugs were considered positives for the myocardial infarction DMEs. The remaining 1474 drugs in our 1970 drug set were considered negatives for the myocardial infarction DME.

### Modeling true positive and negative networks with PathFX

Because we could not create network associations without knowledge of drug-binding targets, we removed drugs without targets in DrugBank, leaving a set of 997 drugs for further analysis. We analyzed these 997 drugs using PathFX with default parameter settings. PathFX uses drug-binding proteins as inputs to identify a protein–protein interaction network around targets based on the likelihood of the interaction existing and then uses network genes/proteins to identify phenotypes enriched in this network relative to the entire interactome. The original interaction network published with PathFX contains an edge score for all protein interactions. The edge score reflects the amount and quality of evidence (e.g. the number of publications, and the type of experimental analysis used to discover the interaction) and all scores are normalized from 0–1. A higher score reflects more and greater quality of evidence that the proteins interact. This scoring is based on the MIScore [34] method and is fully elaborated in [9]. PathFX uses a depth-first search to discover protein–protein interactions around a drugs’ target(s). The depth first search stops when a path score falls below the empirically derived threshold. This path score threshold was derived by measuring path uniqueness per network gene across a wide range of thresholds. At each threshold, and for each gene, the uniqueness of a path was measured as the difference between the path’s score and the average of all path scores for a gene. Path scores greater than the average were considered unique and path scores below the average were considered not unique. The empirical threshold was selected by counting the proportion of total unique paths in the network. At high score thresholds (e.g. 0.99), very few path scores exceeded this threshold and very few paths were unique. As we measured lower values (e.g. 0.7), many more paths were discovered, but the proportion of paths above the average path score for a gene peaked and then diminished. We formulated the scoring this way because highly connected and highly studied genes (e.g. ubiquitin or tumor protein P53 (TP53)) could be compared to their own averages. This would generate a stricter threshold for including highly studied genes without penalizing network genes with fewer interacting partners. In the original PathFX publication, this score was set to 0.77. Unique to our approach; this path score was not optimized for capturing drug–disease associations but was set to minimize biases such as hub bias when including protein interactions in a drug pathway. Conceptually, this path score represented an interaction distance where we had the strongest support from the underlying interactome data.

After identifying a network of proteins, PathFX uses a multiple hypothesis–corrected Fisher’s exact test to measure phenotypes for which network genes are enriched relative to the entire interactome. We calibrated this process by generating networks using random size-matched sets of drug targets and measuring the enrichment to all phenotypes. A real network was considered enriched for a phenotype if the multiple hypothesis–tested *P*-value was above the median *P*-values for 100 random networks seeded with the same number of input proteins. For a full list of features and outputs, see [9, 35]. For each drug, PathFX analysis yielded a PPI network and a list of phenotypes associated with these networks.

When analyzing these 997 drugs, we defined a true positive as a case where PathFX identified the DME, or a synonym, in the drug’s network. We defined a false positive as a case where PathFX identified a DME in the network of a negative drug. We defined a false negative as a case where PathFX didn’t identify a DME associated with a positive drug. We defined a true negative as when PathFX didn’t identify an association to a DME for a negative drug. This analysis was conducted using the script define_tp_fp.py and it created the following outputs: drugs_to_dmes_true_positive.txt, drugs_to_dmes_false_positives.txt, files that contain the true positive and false positive examples. Of the original 34 DMEs, we discovered at least one network association for 24 of the DMEs (Table 2). For remaining analyses, we focused on the 24 DMEs where we found at least one network association.

### Plotting *P*-value distributions and estimating AUROC values

In the same script (define_tp_fp.py) where we defined our true and false positive examples, we generated plots of the *P*-values for these associations. When we started our PathFX algorithm, we hypothesized that an optimized *P*-value threshold may be able to distinguish the true from false positives. However, when we plotted the multiple hypothesis–corrected *P*-values reported from the default PathFX for positive and negative drugs, we generated overlapping distributions. We plotted both the raw *P*-values and the normalized *P*-values. The normalized *P*-values are the multiple hypothesis–corrected *P*-values normalized to the median *P*-value threshold of 100 random networks (explained above). To generate values for the ROC curve, we further filtered the normalized *P*-values for counting true and false positives on a range from 0 to 1 and stored the counts of true positives and false positives for later ROC curves. The define_tp_fp.py script generated the plots, raw_values.png, and norm_pvalues.png, and generated the data object, pvalue_roc_values.pkl, for further analysis. We analyzed the AUROC in the script, plot_ROC_pv_soDist.py, using the trapez method implemented in Python.

### Measuring the effect of interactome distance on detecting DME associations

We developed modified versions of PathFX to test the effect of altering PPI distance on detecting associations to DMEs. Indeed, many successful network approaches have used interactome distance functions to identify relationships between drug targets and disease-associated genes [8, 12, 16]. For the original PathFX construction, we empirically derived an interaction score threshold to prevent hub bias and to maximize path uniqueness (explained above) [9]. A key assumption in these approaches is that a drug’s targets are proximal to DMEor disease-associated genes, or that distance can be described by a single, global function. We hypothesized that changing the path score threshold would reveal an optimal distance to recover relationships between drug target(s) and DME-associated genes. To measure the effect of path distance on detecting associations to side effects, we created 11 custom versions of PathFX each with a different path score threshold. Effectively, changing the score threshold allowed us to focus on networks that were relatively close or far from the drug’s targets. In this case, ‘close’ represented proteins that interacted with drug targets directly and ‘far’ represented proteins that were multiple interactions downstream of a drug target. These scripts are contained in the PathFX_soDist/scripts/directory and are named phenotype_enrichment_pathway_so_dist_0.82.py where ‘0.82’ represents the score threshold used in this version. The other score thresholds used include 0.82–0.90, 0.95 and 0.99. We started our experiment using a stringent, high threshold (i.e. 0.99) and then relaxed this threshold to increasingly allow more edges to be considered in network construction. Given the score distribution of our interaction network, we found that computational time increased significantly as we reached the 0.82 range because consideration of every path relative to all network paths was quite slow. We stopped at this score threshold because the computational time was increasingly slow and because we were not increasing the ability to detect more true positives. In each version, we recalculated the expected distributions of multiple hypothesis–corrected *P*-values per phenotype using 100 random networks with size-matched input sets. A network was associated with a phenotype if the multiple hypothesis–corrected *P*-value was greater than the median *P*-value of 100 random networks for that phenotype with the same number of drug target inputs.

We reanalyzed networks for our 997 drugs at each path score threshold by using each version. Generating networks for all 977 drugs using each of the 11 versions is contained in the script run_pathfx_all_distances.py. As above, we investigated whether the networks for these drugs contained associations to true or false positive DMEs at each score threshold and calculated the sensitivity and specificity as mentioned above. We analyzed these results using the script, count_tp_fp_so_dist.py, and the results of this script are saved in the PathFX_soDist/results/analyze_so_dists/dire ctory. We then used the plot_ROC_pv_soDist.py to count the true and false positive rates at each score threshold and plot the ROC curve using these values. This script generated the pvalue_ROC_dist.png figure.

### Logistic regression, decision trees, and random forests analysis

For this analysis, we created binary matrices for all true and false positive networks associated with a DME. These matrices included a 1/0 if a gene was/was not included in the drug’s network, respectively. Row labels reflected whether the drug’s label was associated with the DME. This analysis is included in the script create_positive_negative_files.ipynb and this analysis yielded a matrix file for each of 24 DMEs: agranulocytosis, cardiac arrest, cerebral infarction, deep vein thrombosis, delirium, edema, gastric ulcer, hemolytic anemia, hemorrhage, hepatic necrosis, hyperlipidemia, hypertension, interstitial lung disease, myocardial infarction, myopathy, pancreatitis, peripheral neuropathy, pneumonia, proteinuria, pulmonary edema, sepsis, tardive dyskinesia, thrombocytopenia, and ventricular tachycardia. To facilitate future analyses, these files are saved in/ML_network_positives_negatives/dme_DMENAME.txt where DMENAME is replaced with each of the DMES of interest.

We first used the scikit-learn module in Python and selected a nested cross-validation procedure to compare logistic regression, decision trees, and random forest because this procedure generated a more realistic estimation of the model generalization capabilities. We used the F1 score to evaluate model performance in this analysis because this metric is robust to imbalances in datasets and because high precision and high recall are valuable for detecting adverse outcomes. We used an inner and outer split of 4 because of the limited size of our dataset and we sampled several parameter configurations for each model type using a grid-based search. Specifically, we tested 1152, 288, and 620 model configurations for random forest, decision trees, and logistic regression, respectively (described more in Table 3). We completed these analyses using the DecisionTreeClassifier, LogisticRegression, and RandomForestClassifer methods from scikit-learn in Python. The results of those analysis are included in Supplemental Fig. 3 and the analysis was completed in the script, ML_network_positives_negatives/run_all_dme_models_ncv.py.

To generate test scores for the ROC curves using logistic regression, we modified ML_network_positives_negati ves/run_all_dme_models_new_log_reg.py and ML_netwo rk_positives_negatives/all_pathways.py scripts. We used default methods in scikit-learn to measure F1, ROC, and accuracy from the logistic regression model. To plot all ROC curves, we used plot_ROC_all_methods_072720.py. We also used default methods in scikit-learn to export the feature importance scores from the LogisticRegression classifier.

### Plotting merged networks

To analyze feature importance scores, we used ML_netwo rk_positives_negatives/save_and_plot_feat_imp_scores.py. This script analyzed the feature importance scores generated after the model fitting and generated Supplemental File 2. This file is a copy of ML_network_positives_negativ es/log_reg/logistic_regression_all_feature_importance.xl sx. We next plotted merged networks and feature importance values using network_images/plot_feat_imp_on_ networks.ipynb. In the Python notebook, we merged interaction networks for positive and negative drugs associated with each DME. Using the merged graph, we created custom methods to plot drugs as triangles in the top-most layer of the graph, drug-binding proteins in the second layer of the graph, intermediate proteins in the third layer of the graph, and DME-related phenotypes in the fourth and bottom layer of the graph. We additionally used the feature importance scores generated previously to create custom color maps for all merged networks highlighting the network proteins with feature importance scores in the logistic regression. This script also generated 16 text files, one for each DME (e.g. ‘edema_feature_import_drug_binding_table.txt’, available in GitHub), from which we created tables associating all network genes to their feature importance scores and node type (either drug binding or not drug binding). We manually copied these files into the SF2_AllDME_feature_import_drug_binding_table_format ted.xlsx to create the Supplemental Table.

### Analyzing features associated with logistic regression performance metrics

We analyzed relationships between input data features and multiple performance metrics. We first calculated the average F-score, average precision, average recall, and AUROC (‘ROC value’), for each DME over a range of acceptance thresholds. This analysis is included in plot_ROC_all_methods_072720.py and used Python Pandas DataFrame objects to generate a table of values for each of the 24 DMEs analyzed with logistic regression. These results are saved in the file DME_individal_ROC_values_input_counts.xls. We further expanded this table to include the total number of genes analyzed, the total number of true positive networks, the total number of false positive networks, the ratio of true to false positive examples, the proportion of true positive examples, the number of singleton genes and the fraction of shared genes between true and false positives per DME. In this case, a ‘singleton’ refers to a gene that is present in only one true or false positive example. We used the script explore_ind_dme_performance.py to add these values to the table. The script generated DME_individual_ROC_values_input_counts_expanded.xls. These values are also included in Supplemental Table 2. To calculate correlation values, we used the CORREL function in Excel and exported a formatted table to illustrate the range of values. This formatted table is included in the GitHub repository as DME_individual_ROC_values_ input_counts_annotated.xlsx.

## Supplementary Material

Supp_File_2

Supp_File_1

Supplementary data

Supplementary data are available at *INTBIO Journal* online.

## Figures and Tables

**Table 1.** Average precision per approach.

	*P*-value threshold	Distance	CSI
Average precision	0.284	0.256	0.499

**Table 2.** Number of true positives identified in PathFX networks by DME.

DME	Number of positives

Hypertension	841
Myocardial infarction	263
Hemorrhage	154
Pancreatitis	128
Pneumonia	125
Edema	54
Myopathy	36
Tardive dyskinesia	35
Hyperlipidemia	35
Sepsis	29
Peripheral neuropathy	28
Thrombocytopenia	27
Proteinuria	27
Gastric ulcer	26
Pulmonary edema	18
Delirium	17
Cerebral infarction	10
Hemolytic anemia	9
Cardiac arrest	7
Hepatic necrosis	4
Agranulocytosis	5
Ventricular tachycardia	2
Interstitial lung disease	2
Deep vein thrombosis	1

**Table 3.** Model configurations explored during nested cross-validation.

Method	Features	Total configurations

Random forest	n_estimators: 10, 100, 200, 300, 400, 500, 600, or 700; criterion: gini or entropy; max_depth: no limit, 3, 8, or 13; min_samples_split: 2, 10, or 30; min_samples_leaf: 1, 10, or 20; class_weight: balanced or no weights	1152
Decision trees	criterion: gini or entropy; max_depth: no limit, 3, 8, or 13; min_samples_split: 2, 10, or 30; min_samples_leaf: 1, 10, or 20; class_weight: balanced or no weights; splitter: best or random	288
Logistic regression	penalty: L1 or L2 C: the concatenation of numbers spaced evenly on a log scale starting from −5 to 4 generated from 10 samples and from 300 samples	620

Total configurations resulted from the Cartesian product of each feature vector.

## Data Availability

The data and code used in this analysis are available on GitHub (https://github.com/jenwilson521/network_selection).

## INSIGHT

Drug side effects are routinely analyzed using target proteins, yet targets influence protein networks. We studied severe side effects and discovered that downstream proteins were often more predictive of drug side effects than targets, suggesting that downstream protein analysis could enhance side effect prediction. Our approach is innovative because we inferred a set of noncausative drugs using drug label data, and we emphasized a per-side effect approach; many approaches apply global rules for predicting drug network effects. We benefitted from the integration of drug targets, protein networks, and side effect phenotype data to discover the predictive power of downstream proteins.
