# Supplementary material for: From random to predictive: a context-specific interaction framework improves selection of drug protein–protein interactions for unknown drug pathways
Source: Integr Biol (Camb). Author manuscript; Available in PMC 2026 Jun 15. (PMC13265242; doi:10.1093/intbio/zyac002)
Supplement: Supp_File_1 [file NIHMS2168077-supplement-Supp_File_1.pdf]

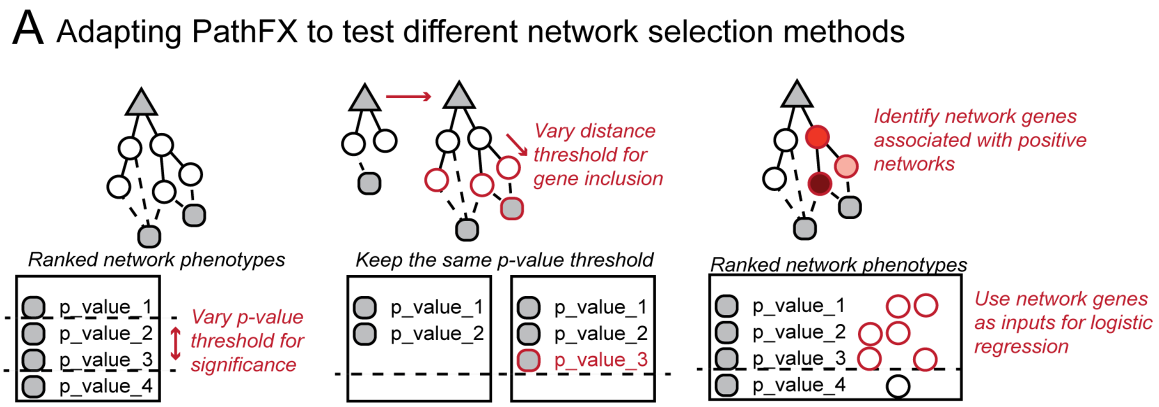

Supplemental Figure 1. **Adaptation of PathFX for testing of network frameworks.** (**A**) To test various theoretical approaches, we adapted PathFX in three ways: first we constructed networks using the optimal distance threshold derived in (9) and then varied the p-value threshold used to select associations discovered by PathFX (left). Next we generated multiple versions of PathFX using a range of distances to explore whether a stringent or relaxed search of the network would correctly associate a drug's targets to relevant DMEs (middle). Lastly, we used PathFX with the distance and p-value thresholds derived in (9) and then analyzed DME-associated network genes with subsequent machine learning analysis (right). In all cases, PathFX yielded an interaction network and a table of ranked phenotype associations. For all diagrams, drugs, network proteins, and phenotypes are represented as triangles, circles, and rounded squares.

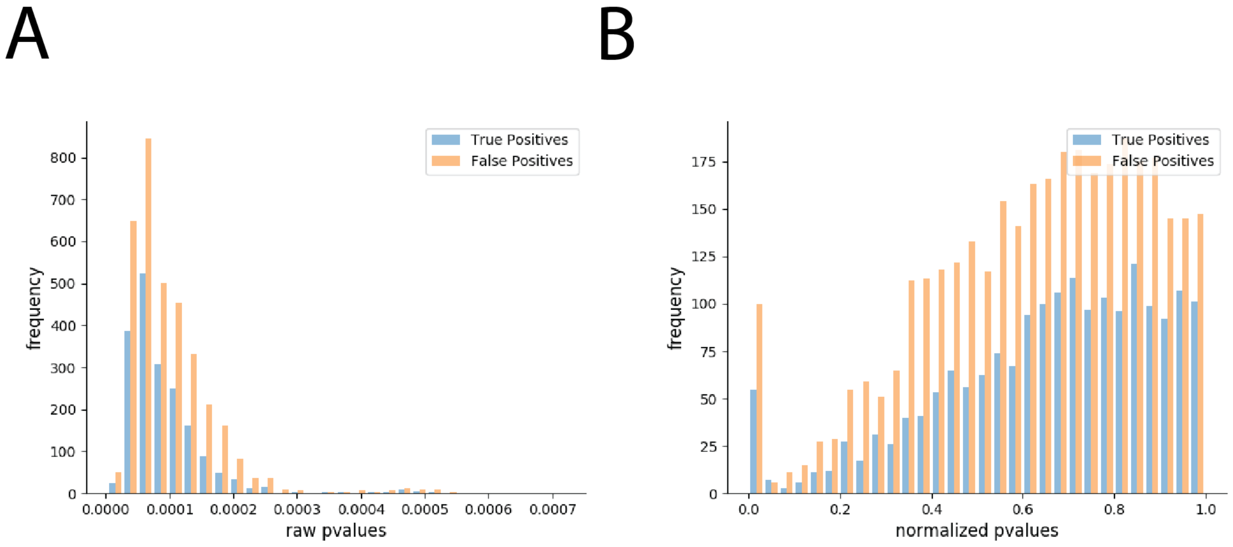

Supplemental Figure 2. **Comparison of raw and normalized p-values for true positives and false positives**. (**A**) Distribution of p-values for true and false positive drug associations to DMEs. (**B**) Distribution of normalized p-values for true and false positive drug associations to DMEs. In PathFX, a p-value threshold is derived for each phenotype in the algorithm to prevent study-bias. For instance, the phenotype “cancer” is associated to many network genes and thus a higher level of significance is required to include “cancer” in a drug’s network. This threshold was derived by generating random networks and then measuring the distribution of scores for each phenotype. The normalized p-value represents a network association scaled to the median p-value discovered from random networks and this process is repeated per phenotype and per number of drug target inputs.

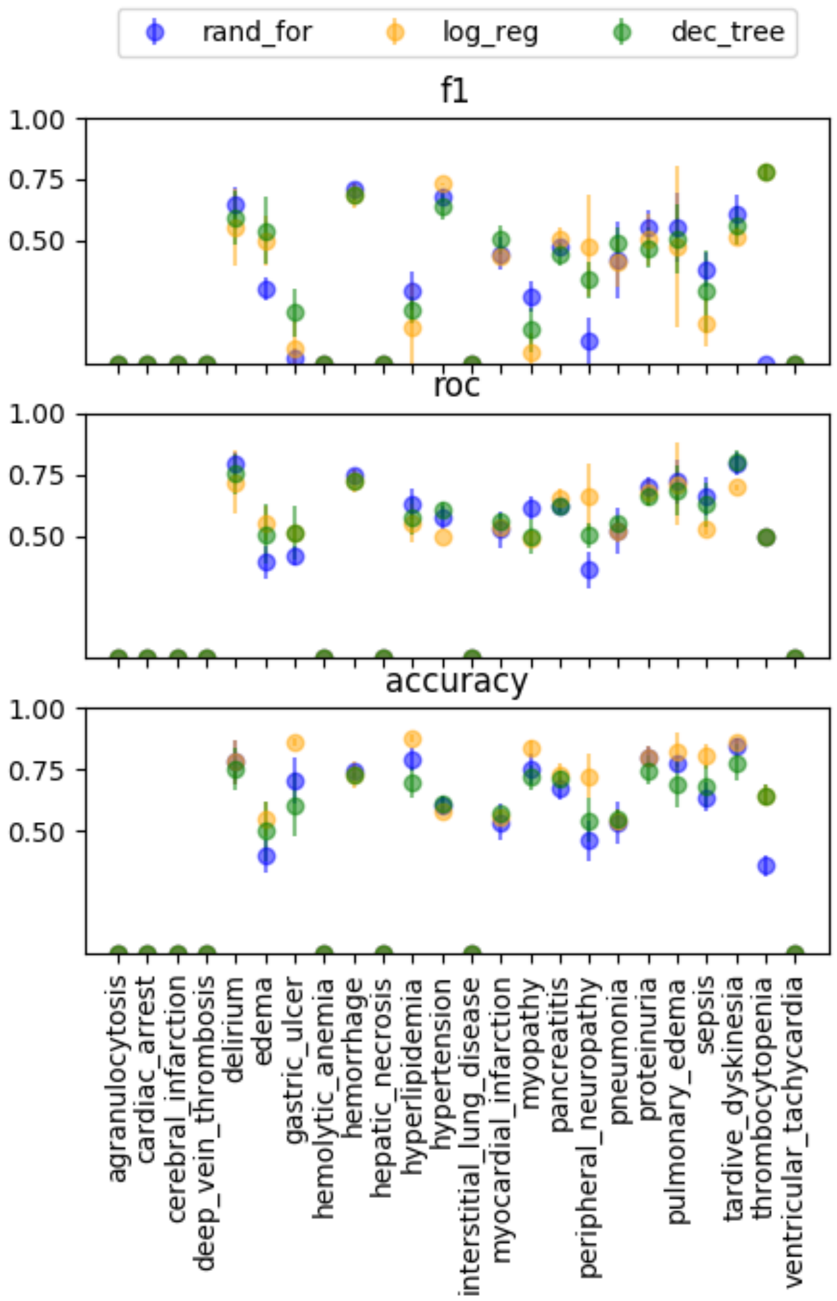

Supplemental Figure 3. Performance of three modeling approaches using nested cross validation analysis. We applied a nested cross validation approach using decision trees (green), random forests (blue), and logistic regression (orange) for 16 DMEs. The remaining 8 DMEs were skipped because they had fewer than 10 positive or 10 negative cases and we considered this to be insufficient data for creating a model. We assessed model performance by F1, ROC, and Accuracy scores. Error bars represent the standard deviation of 500 splits of the data.

	ROC	F1	Accuracy
dec_tree	0.6042	0.4639	0.6560
rand_for	0.5983	0.4321	0.6487
log_reg	0.5954	0.4380	0.7297

Supplemental Table 1. Average ROC, F1, and Accuracy scores for DMEs used in nested cross-validation. Scores were averaged across the 16 DME models tested. As described in Supplementary Figure 1, we did not perform model assessment for any cases where we had fewer than 10 positive or fewer than 10 negative cases.

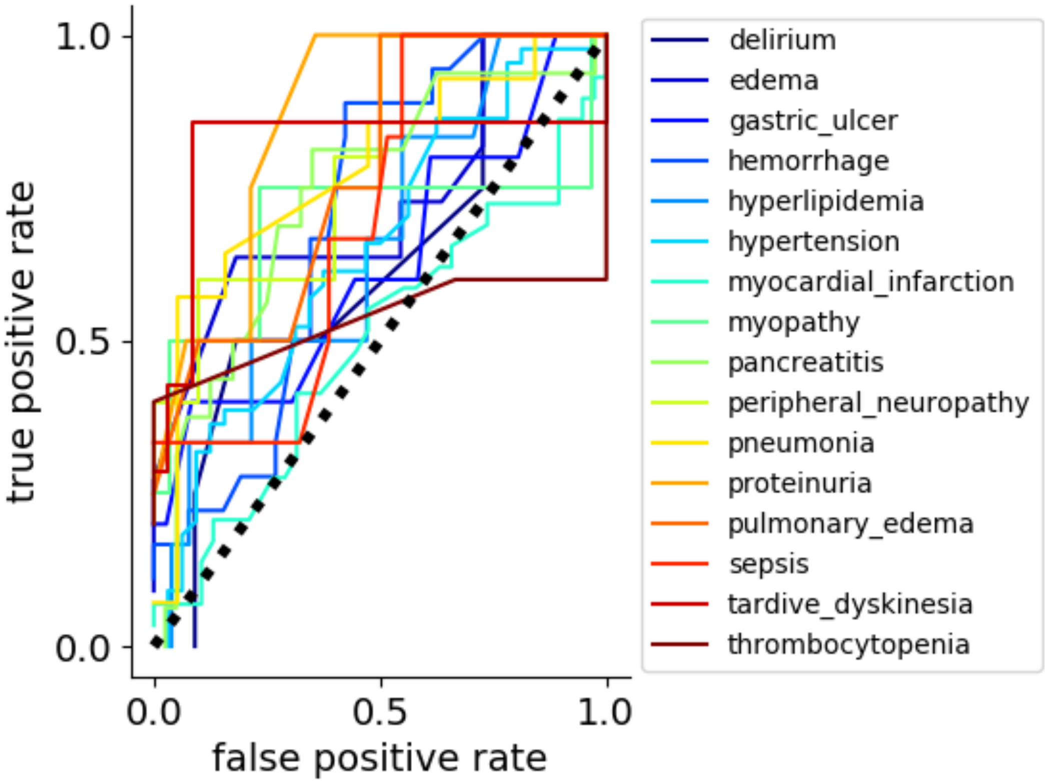

Supplemental Figure 4. ROC curves for all DMEs. ROC curves are plotted for all 16 DME models.

A

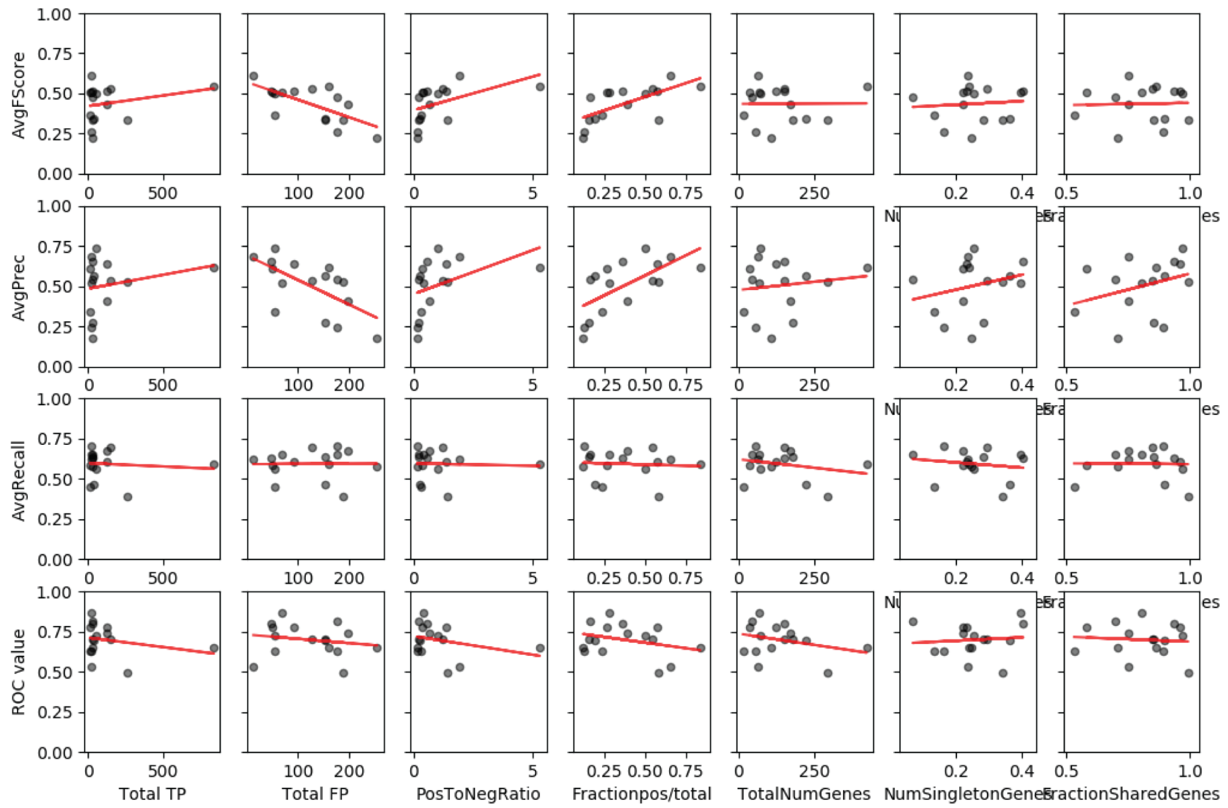

B

	True Positive Count	False Positive Count	Ratio	Fraction pos/total	Total Num Genes	Num Singleton Genes	Fraction Shared Genes
Avg Fscore	0.239	-0.677	0.475	0.661	0.008	0.088	0.034
Avg Prec	0.208	-0.628	0.412	0.643	0.130	0.251	0.319
Avg Recall	-0.095	0.012	-0.041	-0.075	-0.255	-0.167	-0.015
AUROC	-0.243	-0.185	-0.296	-0.307	-0.301	0.093	-0.083

Supplemental Figure 5. Performance values plotted against dataset features per DME. The average F-score, average precision, average recall, and area under the ROC curve (“ROC value”) are plotted against the total number of true positives (“Total TP”), total number of false positives (“Total FP”), ratio of number of positives to number of false positives (“PosToNegRatio”), fraction of all cases that are true positives (“Fractionpos/total”), total number of genes (“TotalNumGenes’), number of genes appearing in only a single network (“NumSingletonGenes”), and the fraction of genes that are shared between true and false positive drug networks (“FractionSharedGenes”) (**A**). Each dot represents an individual DME and the red line represents a least-squares fit line. The correlation between each variable is provided in (**B**). Darker blue and brighter orange reflect greater negative or positive correlation scores relative to all correlations.

name	Avg FScore	Avg Prec	Avg Recall	ROC value	Total FP	Total TP	Pos To Neg Ratio	Fraction pos/total	Total Num Genes	Num Singleton Genes	Fraction Shared Genes
delirium	0.364	0.337	0.453	0.625	55.000	17.000	0.309	0.236	15.000	0.133	0.533
edema	0.498	0.739	0.565	0.723	54.000	54.000	1.000	0.500	71.000	0.254	0.972
gastric_ulcer	0.257	0.240	0.702	0.625	176.000	26.000	0.148	0.129	55.000	0.164	0.891
hemorrhage	0.533	0.538	0.698	0.702	126.000	154.000	1.222	0.550	150.000	0.293	0.847
hyperlipidemia	0.221	0.173	0.580	0.652	253.000	35.000	0.138	0.122	109.000	0.248	0.706
hypertension	0.543	0.614	0.593	0.648	159.000	841.000	5.289	0.841	421.000	0.240	0.865
myocardial_infarction	0.338	0.528	0.389	0.495	187.000	263.000	1.406	0.584	292.000	0.342	0.993
myopathy	0.343	0.565	0.463	0.692	152.000	36.000	0.237	0.191	223.000	0.363	0.897
pancreatitis	0.430	0.409	0.675	0.740	197.000	128.000	0.650	0.394	172.000	0.221	0.750
peripheral_neuropathy	0.511	0.655	0.628	0.800	49.000	28.000	0.571	0.364	151.000	0.404	0.934
pneumonia	0.517	0.643	0.609	0.778	92.000	125.000	1.359	0.576	124.000	0.234	0.960
proteinuria	0.506	0.516	0.650	0.866	68.000	27.000	0.397	0.284	66.000	0.394	0.803
pulmonary_edema	0.504	0.608	0.583	0.775	50.000	18.000	0.360	0.265	36.000	0.222	0.583
sepsis	0.336	0.273	0.638	0.702	152.000	29.000	0.191	0.160	179.000	0.285	0.855
tardive_dyskinesia	0.475	0.544	0.651	0.816	175.000	35.000	0.200	0.167	43.000	0.070	0.698
thrombocytopenia	0.609	0.685	0.620	0.533	14.000	27.000	1.929	0.659	64.000	0.234	0.750

Supplemental Table 2. Input data features and performance metrics per DME.

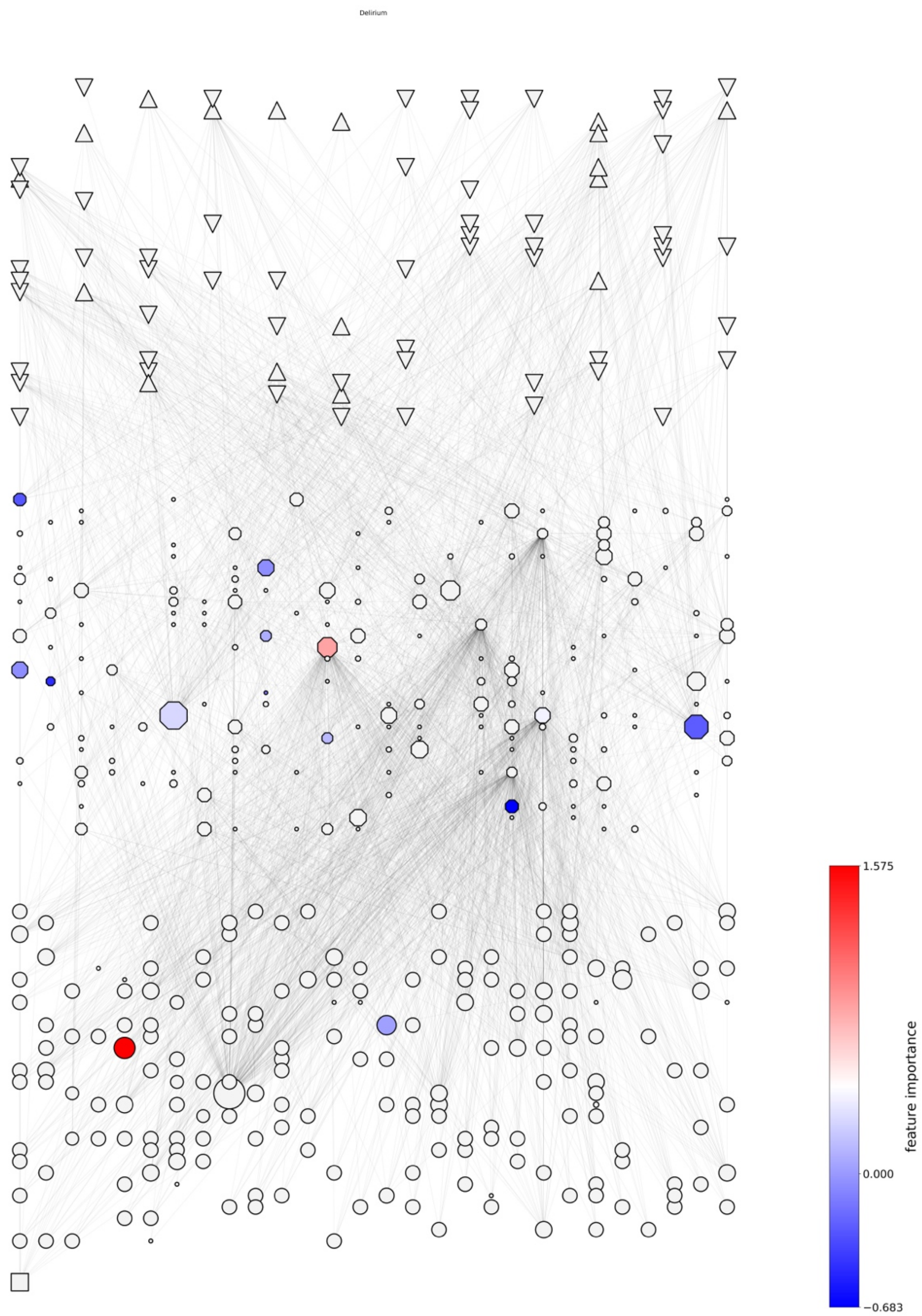

Supplemental Figure 6. Delirium – merged network and feature importance values. The merged interaction network for all true and false positive drugs associated with delirium highlights which network components – drug-binding and network proteins – have high feature importance in the logistic regression model. True/false positive drugs are represented in the top layer as regular/inverted triangles respectively. Drug-binding and intermediate pathway proteins are represented in the second and third layers. The size of the protein reflects the number of networks in which the protein appears. Relevant delirium-associated phenotypes

are represented as boxes in the last layer. Protein coloring reflects the feature importance in the logistic regression model. Red/blue coloring represents association to true/false positive networks.

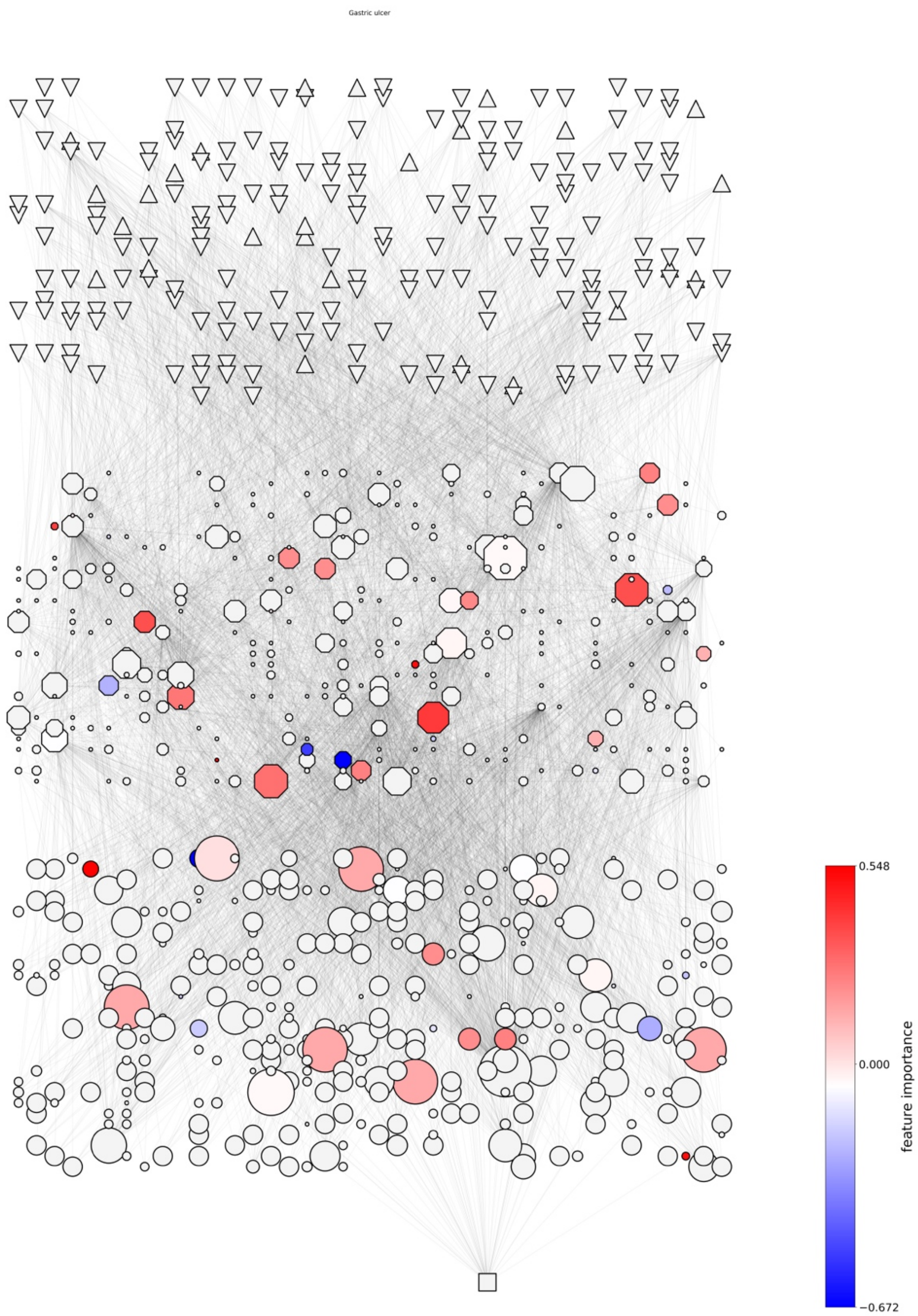

Supplemental Figure 7. Gastric ulcer – merged network and feature importance values. The merged interaction network for all true and false positive drugs associated with gastric ulcer highlights which network

components – drug-binding and network proteins – have high feature importance in the logistic regression model. True/false positive drugs are represented in the top layer as regular/inverted triangles respectively. Drug-binding and intermediate pathway proteins are represented in the second and third layers. The size of the protein reflects the number of networks in which the protein appears. Relevant gastric ulcer-associated phenotypes are represented as boxes in the last layer. Protein coloring reflects the feature importance in the logistic regression model. Red/blue coloring represents association to true/false positive networks.

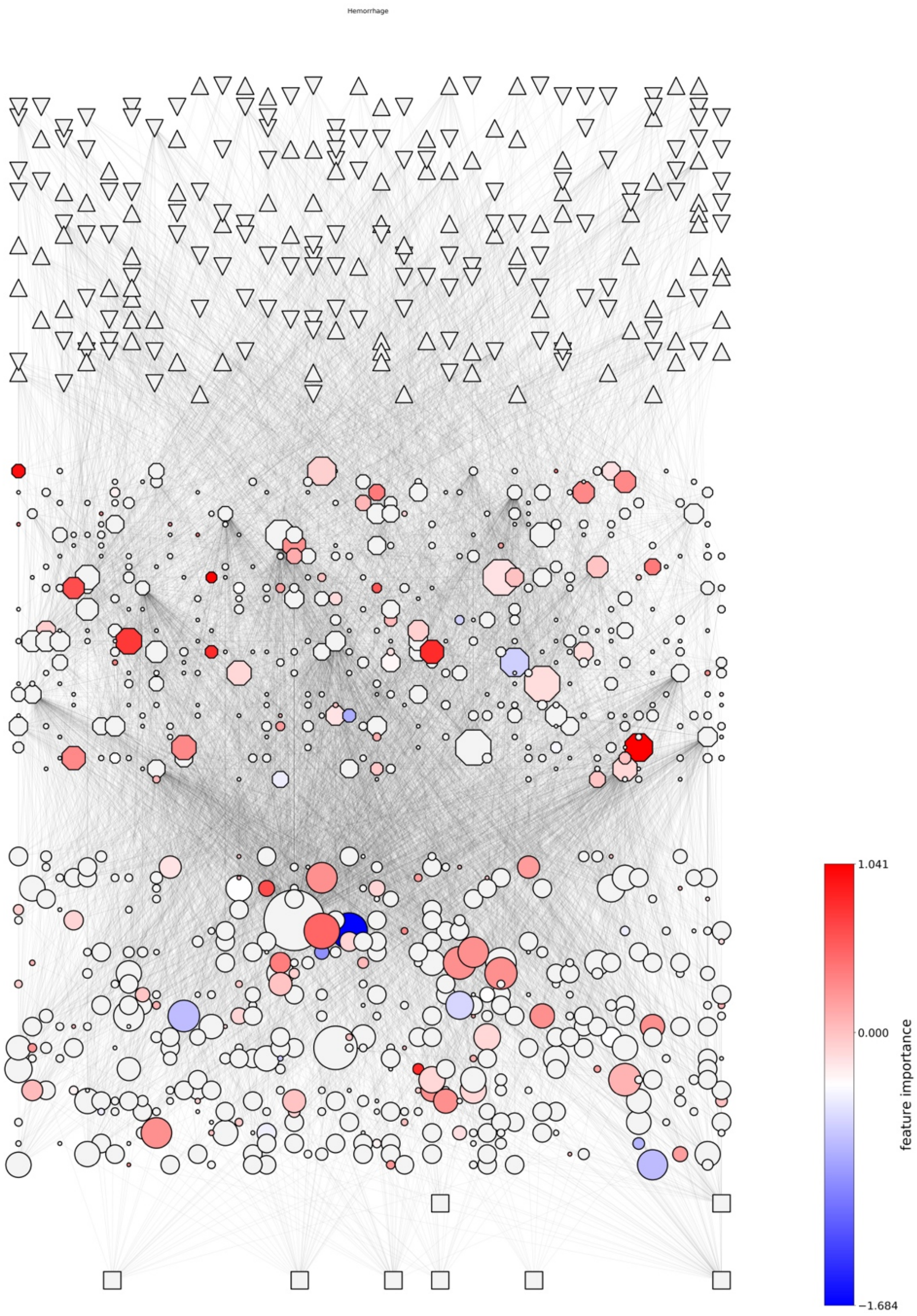

Supplemental Figure 8. Hemorrhage – merged network and feature importance values. The merged interaction network for all true and false positive drugs associated with hemorrhage highlights which network components – drug-binding and network proteins – have high feature importance in the logistic regression model. True/false positive drugs are represented in the top layer as regular/inverted triangles respectively. Drug-binding and intermediate pathway proteins are represented in the second and third layers. The size of the

protein reflects the number of networks in which the protein appears. Relevant hemorrhage-associated phenotypes are represented as boxes in the last layer. Protein coloring reflects the feature importance in the logistic regression model. Red/blue coloring represents association to true/false positive networks.

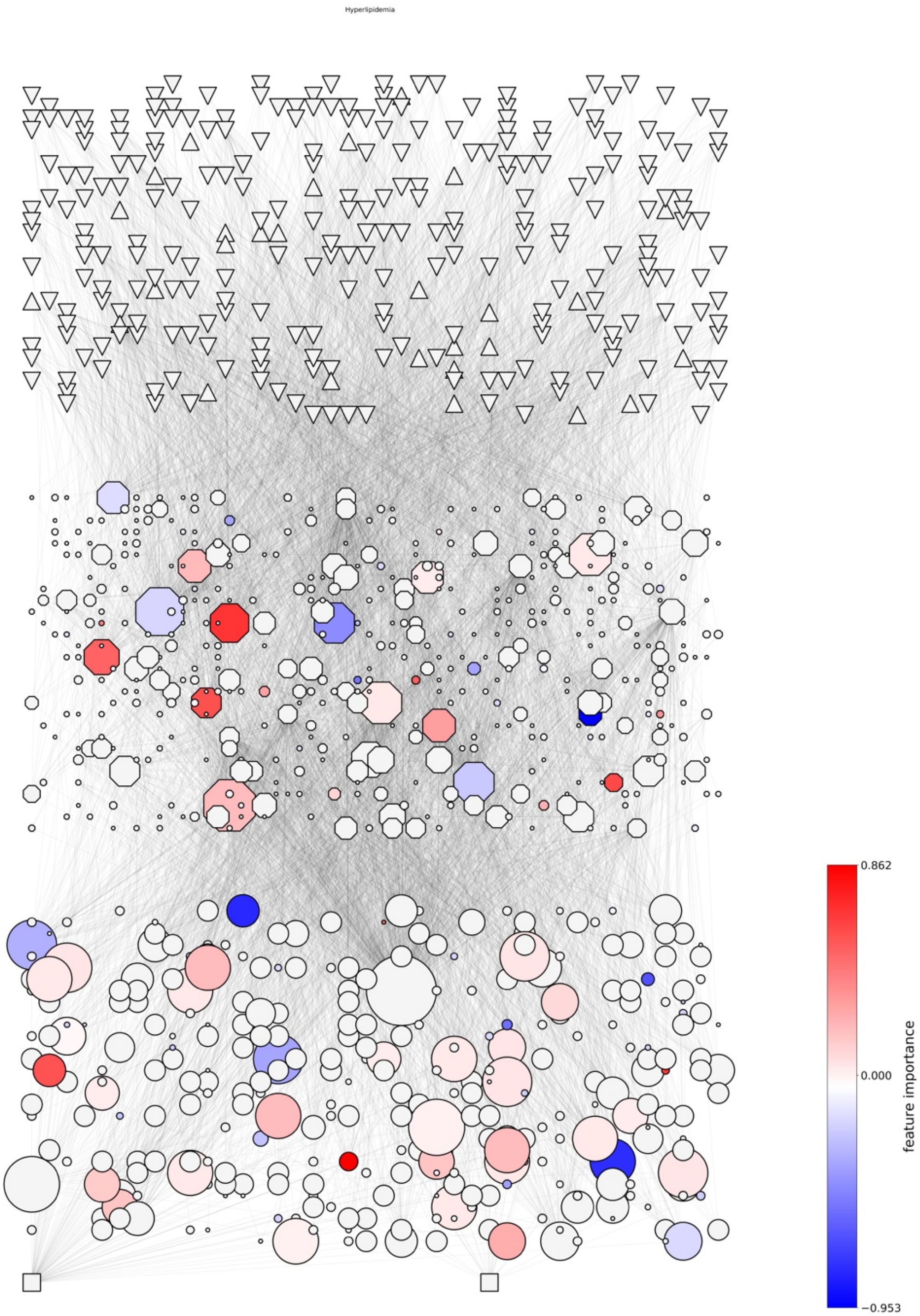

Supplemental Figure 9. Hyperlipidemia – merged network and feature importance values. The merged interaction network for all true and false positive drugs associated with hyperlipidemia highlights which network components – drug-binding and network proteins – have high feature importance in the logistic regression model. True/false positive drugs are represented in the top layer as regular/inverted triangles respectively. Drug-binding and intermediate pathway proteins are represented in the second and third layers. The size of the protein reflects the number of networks in which the protein appears. Relevant hyperlipidemia-associated phenotypes are represented as boxes in the last layer. Protein coloring reflects the feature importance in the logistic regression model. Red/blue coloring represents association to true/false positive networks.

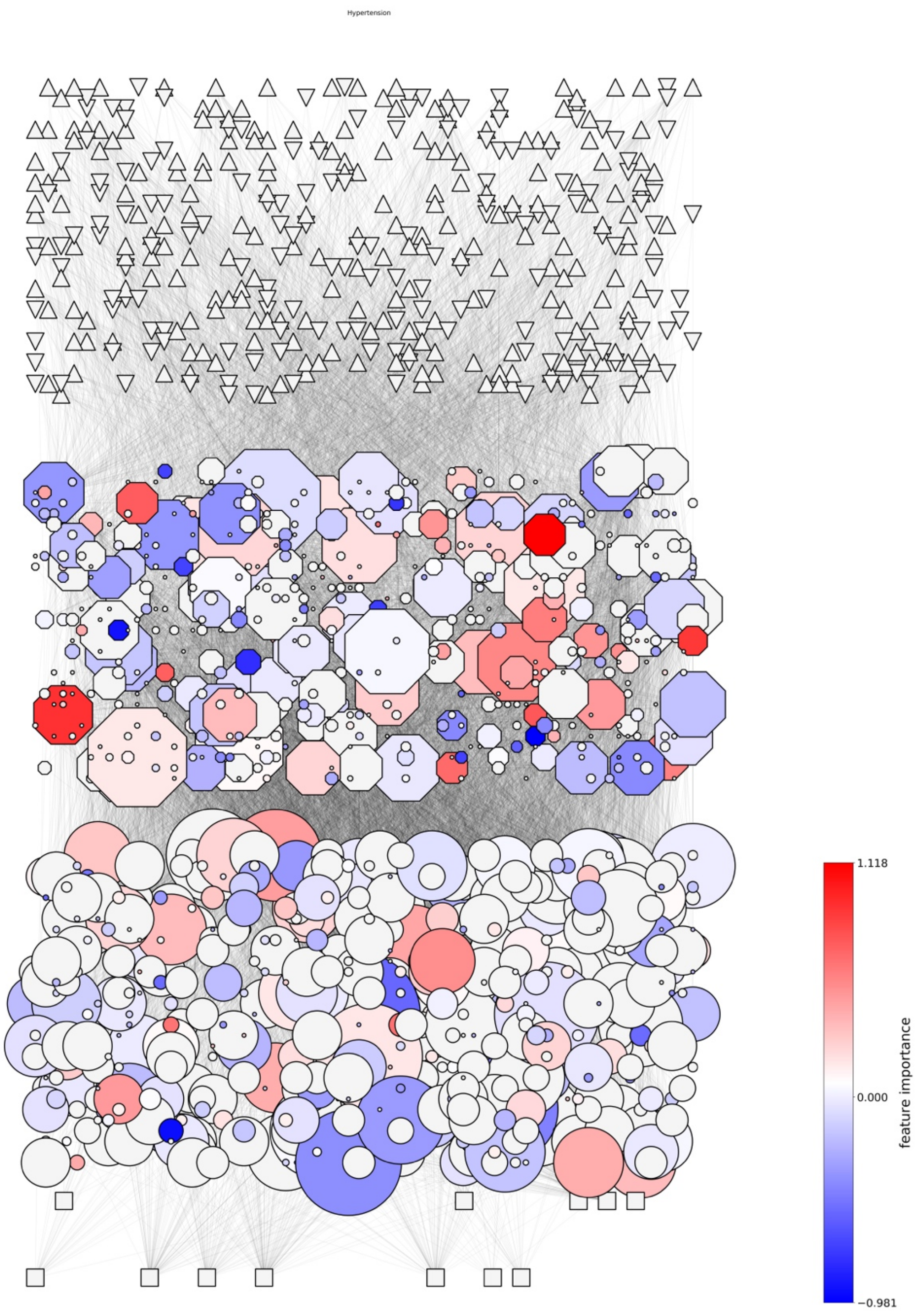

Supplemental Figure 10. Hypertension – merged network and feature importance values. The merged interaction network for all true and false positive drugs associated with hypertension highlights which network components – drug-binding and network proteins – have high feature importance in the logistic regression model. True/false positive drugs are represented in the top layer as regular/inverted triangles respectively. Drug-binding and intermediate pathway proteins are represented in the second and third layers. The size of the

protein reflects the number of networks in which the protein appears. Relevant hypertension-associated phenotypes are represented as boxes in the last layer. Protein coloring reflects the feature importance in the logistic regression model. Red/blue coloring represents association to true/false positive networks.

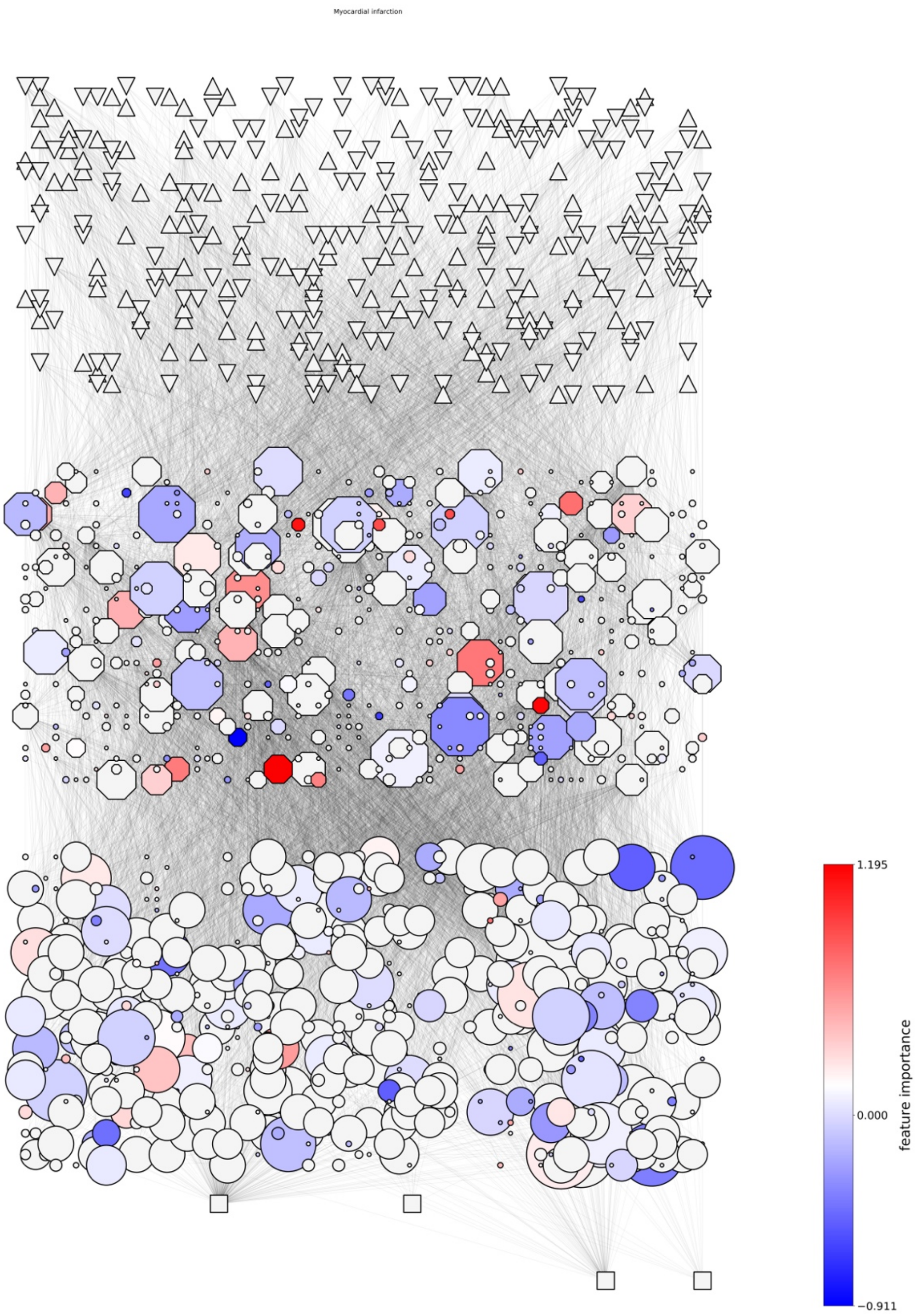

Supplemental Figure 11. Myocardial infarction – merged network and feature importance values. The merged interaction network for all true and false positive drugs associated with myocardial infarction highlights

which network components – drug-binding and network proteins – have high feature importance in the logistic regression model. True/false positive drugs are represented in the top layer as regular/inverted triangles respectively. Drug-binding and intermediate pathway proteins are represented in the second and third layers. The size of the protein reflects the number of networks in which the protein appears. Relevant myocardial infarction-associated phenotypes are represented as boxes in the last layer. Protein coloring reflects the feature importance in the logistic regression model. Red/blue coloring represents association to true/false positive networks.

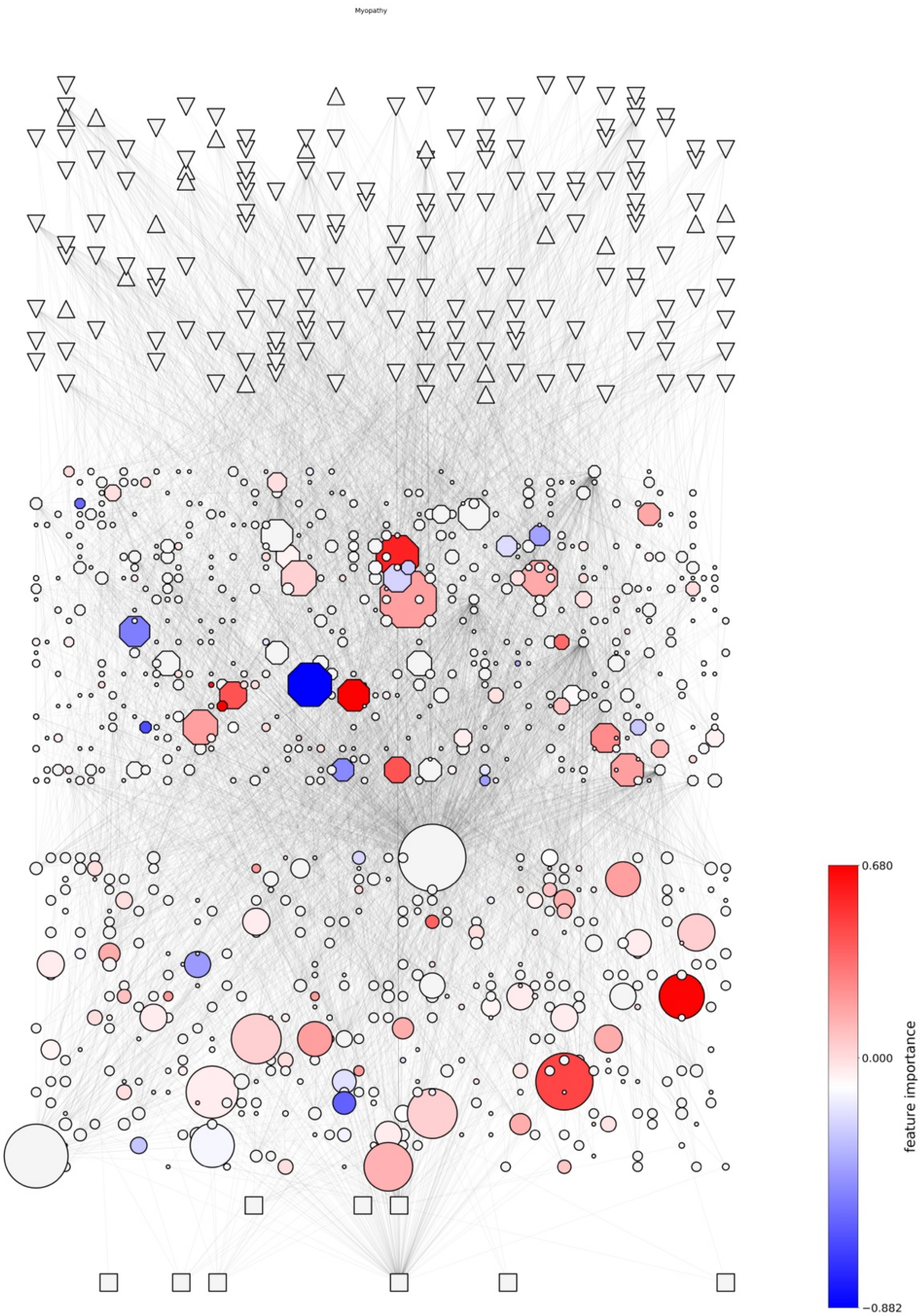

Supplemental Figure 12. Myopathy – merged network and feature importance values. The merged interaction network for all true and false positive drugs associated with myopathy highlights which network components – drug-binding and network proteins – have high feature importance in the logistic regression model. True/false positive drugs are represented in the top layer as regular/inverted triangles respectively.

Drug-binding and intermediate pathway proteins are represented in the second and third layers. The size of the protein reflects the number of networks in which the protein appears. Relevant myopathy-associated phenotypes are represented as boxes in the last layer. Protein coloring reflects the feature importance in the logistic regression model. Red/blue coloring represents association to true/false positive networks.

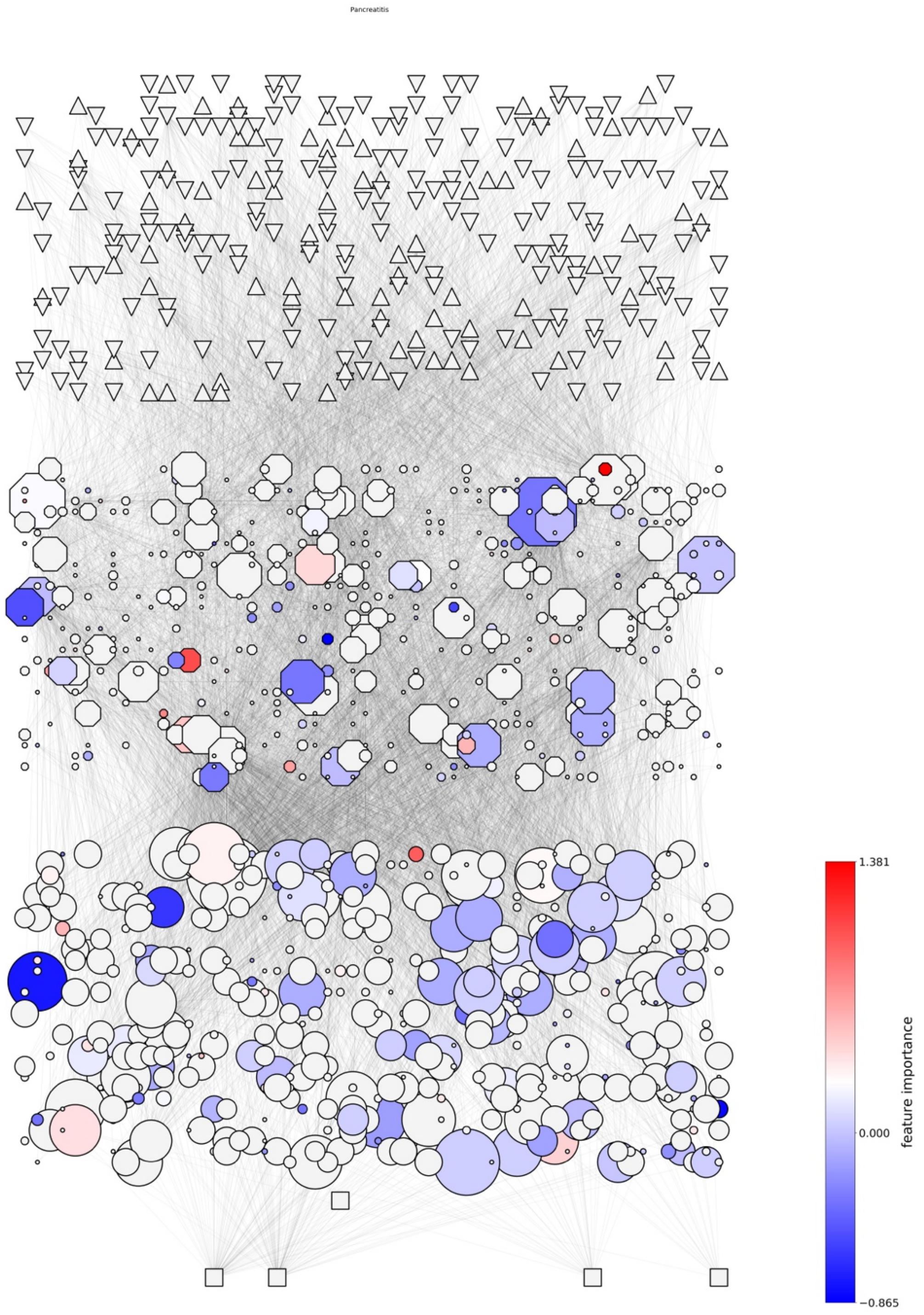

Supplemental Figure 13. Pancreatitis – merged network and feature importance values. The merged interaction network for all true and false positive drugs associated with pancreatitis highlights which network components – drug-binding and network proteins – have high feature importance in the logistic regression model. True/false positive drugs are represented in the top layer as regular/inverted triangles respectively. Drug-binding and intermediate pathway proteins are represented in the second and third layers. The size of the protein reflects the number of networks in which the protein appears. Relevant pancreatitis-associated phenotypes are represented as boxes in the last layer. Protein coloring reflects the feature importance in the logistic regression model. Red/blue coloring represents association to true/false positive networks.

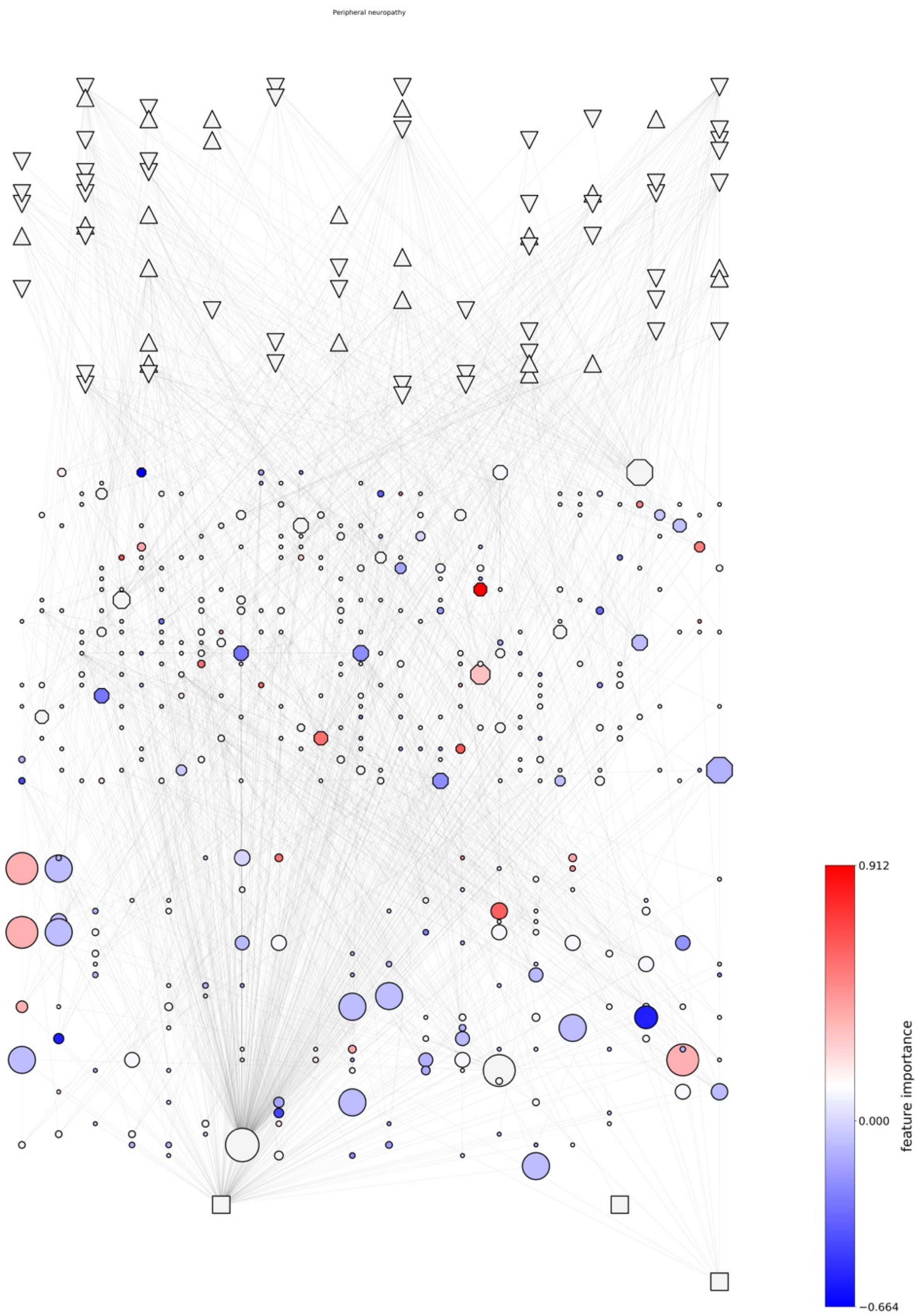

Supplemental Figure 14. Peripheral neuropathy – merged network and feature importance values. The merged interaction network for all true and false positive drugs associated with peripheral neuropathy highlights which network components – drug-binding and network proteins – have high feature importance in the logistic regression model. True/false positive drugs are represented in the top layer as regular/inverted triangles respectively. Drug-binding and intermediate pathway proteins are represented in the second and third

layers. The size of the protein reflects the number of networks in which the protein appears. Relevant peripheral neuropathy-associated phenotypes are represented as boxes in the last layer. Protein coloring reflects the feature importance in the logistic regression model. Red/blue coloring represents association to true/false positive networks.

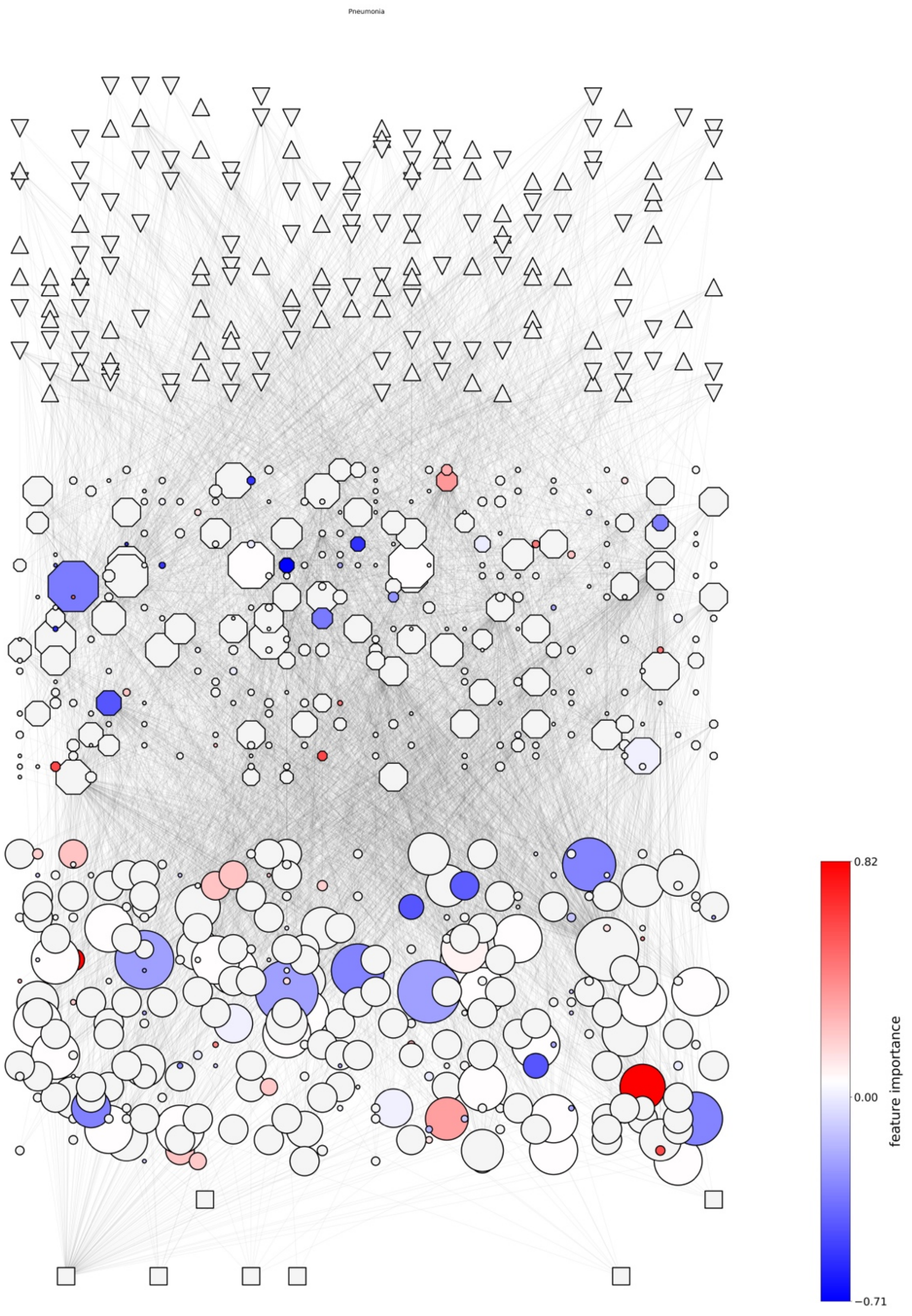

Supplemental Figure 15. Pneumonia – merged network and feature importance values. The merged interaction network for all true and false positive drugs associated with pneumonia highlights which network components – drug-binding and network proteins – have high feature importance in the logistic regression model. True/false positive drugs are represented in the top layer as regular/inverted triangles respectively. Drug-binding and intermediate pathway proteins are represented in the second and third layers. The size of the protein reflects the number of networks in which the protein appears. Relevant pneumonia-associated phenotypes are represented as boxes in the last layer. Protein coloring reflects the feature importance in the logistic regression model. Red/blue coloring represents association to true/false positive networks.

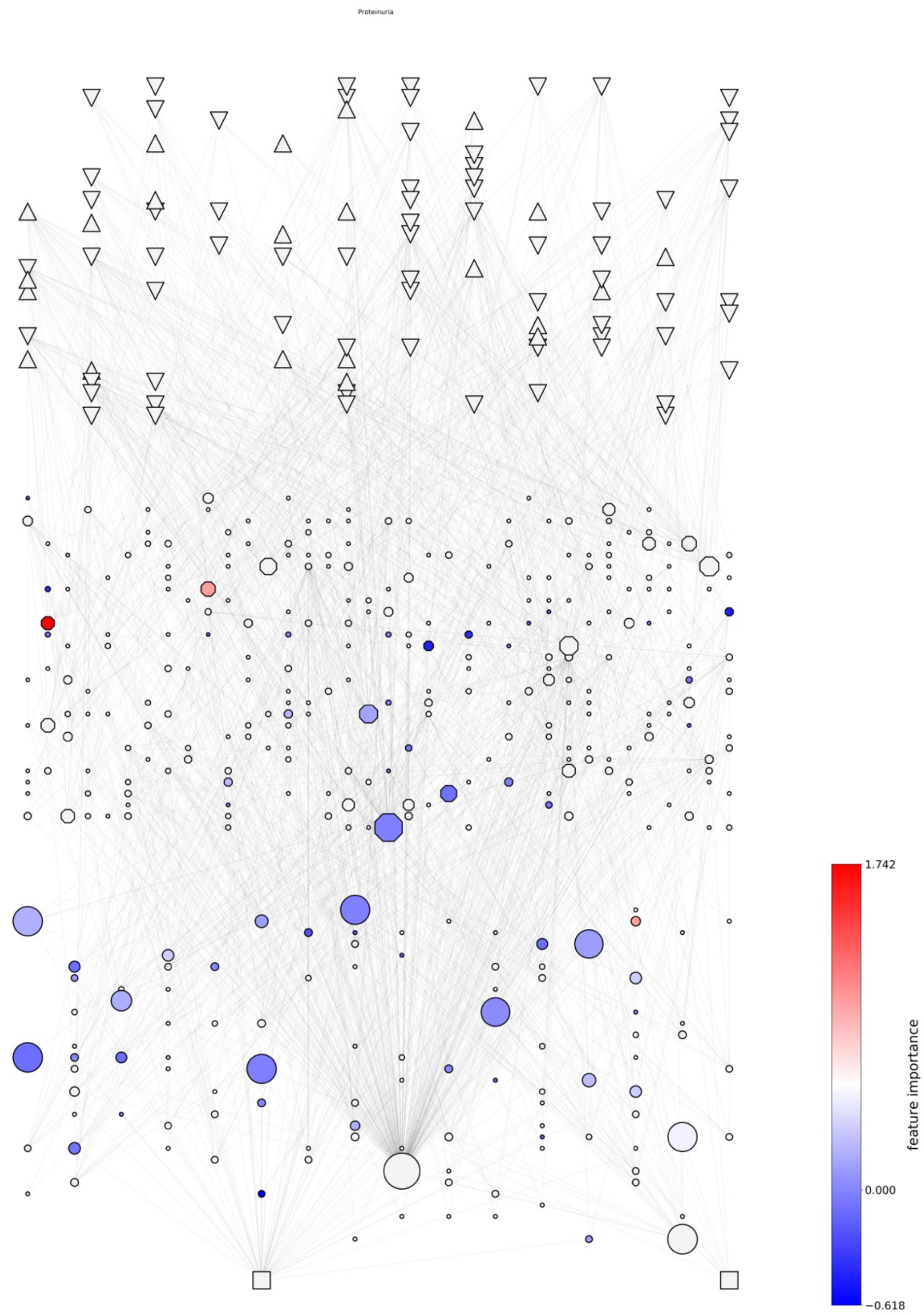

Supplemental Figure 16. Proteinuria – merged network and feature importance values. The merged interaction network for all true and false positive drugs associated with proteinuria highlights which network components – drug-binding and network proteins – have high feature importance in the logistic regression model. True/false positive drugs are represented in the top layer as regular/inverted triangles respectively. Drug-binding and intermediate pathway proteins are represented in the second and third layers. The size of the

protein reflects the number of networks in which the protein appears. Relevant proteinuria-associated phenotypes are represented as boxes in the last layer. Protein coloring reflects the feature importance in the logistic regression model. Red/blue coloring represents association to true/false positive networks.

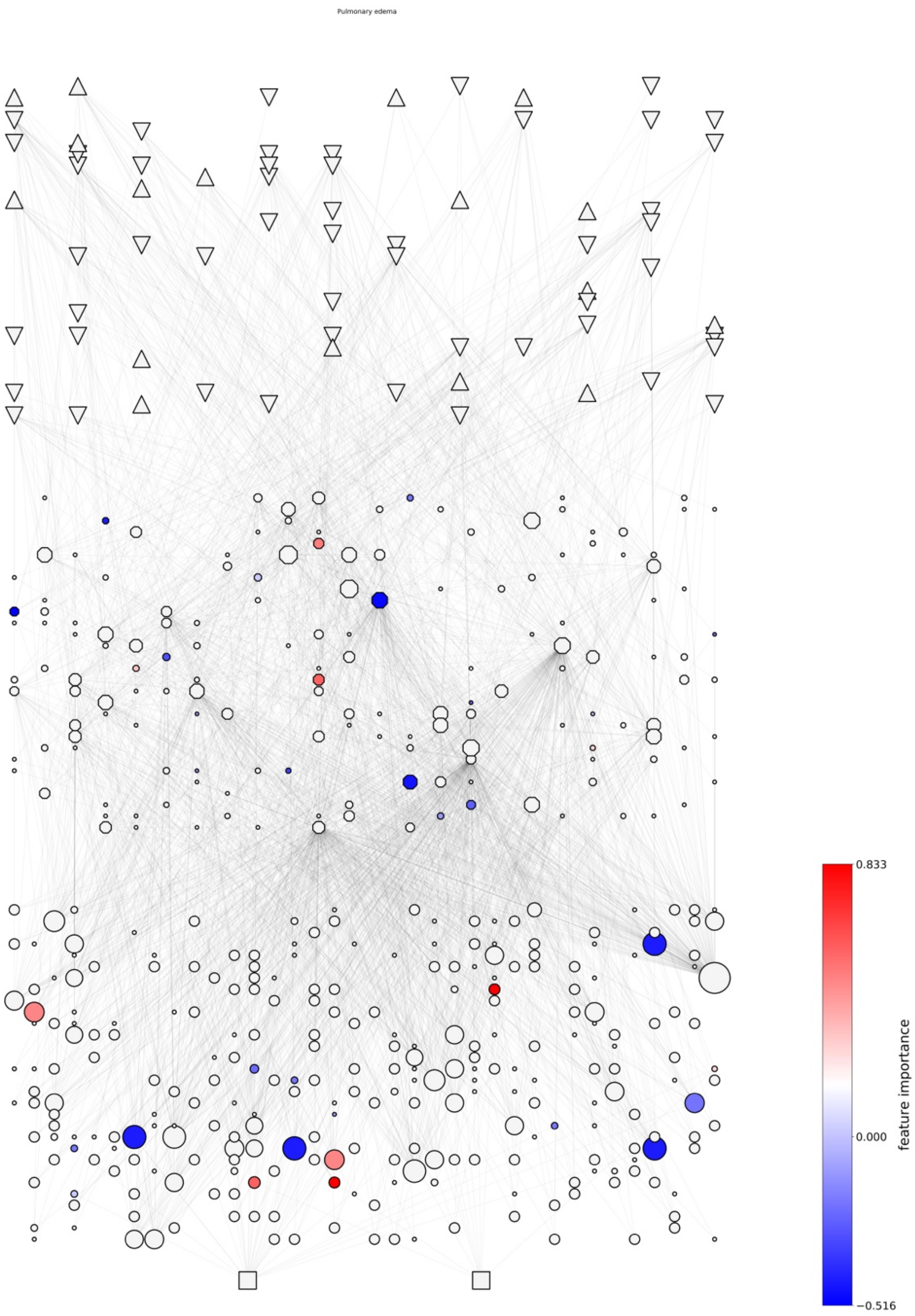

Supplemental Figure 17. Pulmonary edema – merged network and feature importance values. The merged interaction network for all true and false positive drugs associated with pulmonary edema highlights

which network components – drug-binding and network proteins – have high feature importance in the logistic regression model. True/false positive drugs are represented in the top layer as regular/inverted triangles respectively. Drug-binding and intermediate pathway proteins are represented in the second and third layers. The size of the protein reflects the number of networks in which the protein appears. Relevant pulmonary edema-associated phenotypes are represented as boxes in the last layer. Protein coloring reflects the feature importance in the logistic regression model. Red/blue coloring represents association to true/false positive networks.

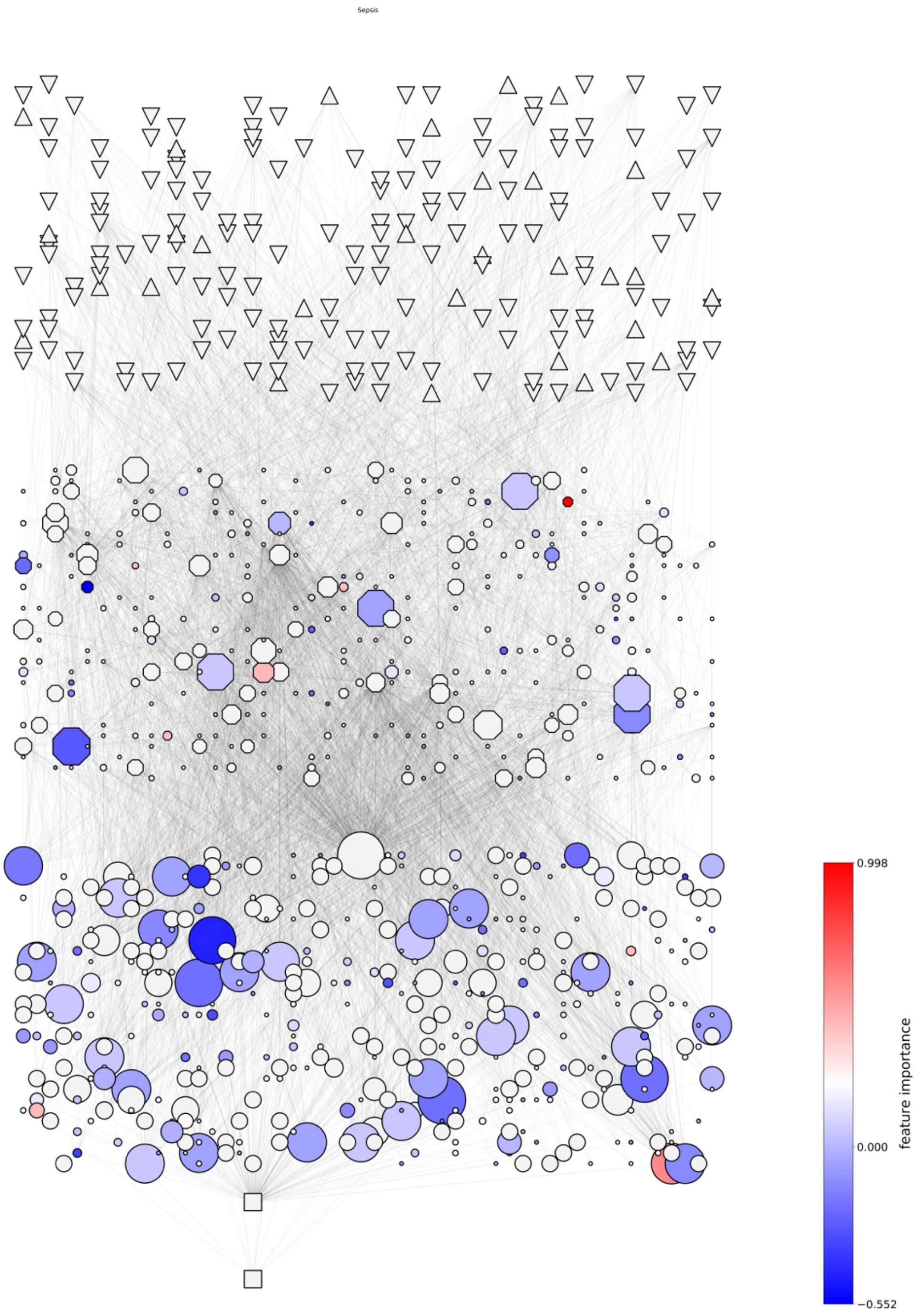

Supplemental Figure 18. Sepsis – merged network and feature importance values. The merged interaction network for all true and false positive drugs associated with sepsis highlights which network components – drug-binding and network proteins – have high feature importance in the logistic regression model. True/false positive drugs are represented in the top layer as regular/inverted triangles respectively. Drug-binding and intermediate pathway proteins are represented in the second and third layers. The size of the

protein reflects the number of networks in which the protein appears. Relevant sepsis-associated phenotypes are represented as boxes in the last layer. Protein coloring reflects the feature importance in the logistic regression model. Red/blue coloring represents association to true/false positive networks.

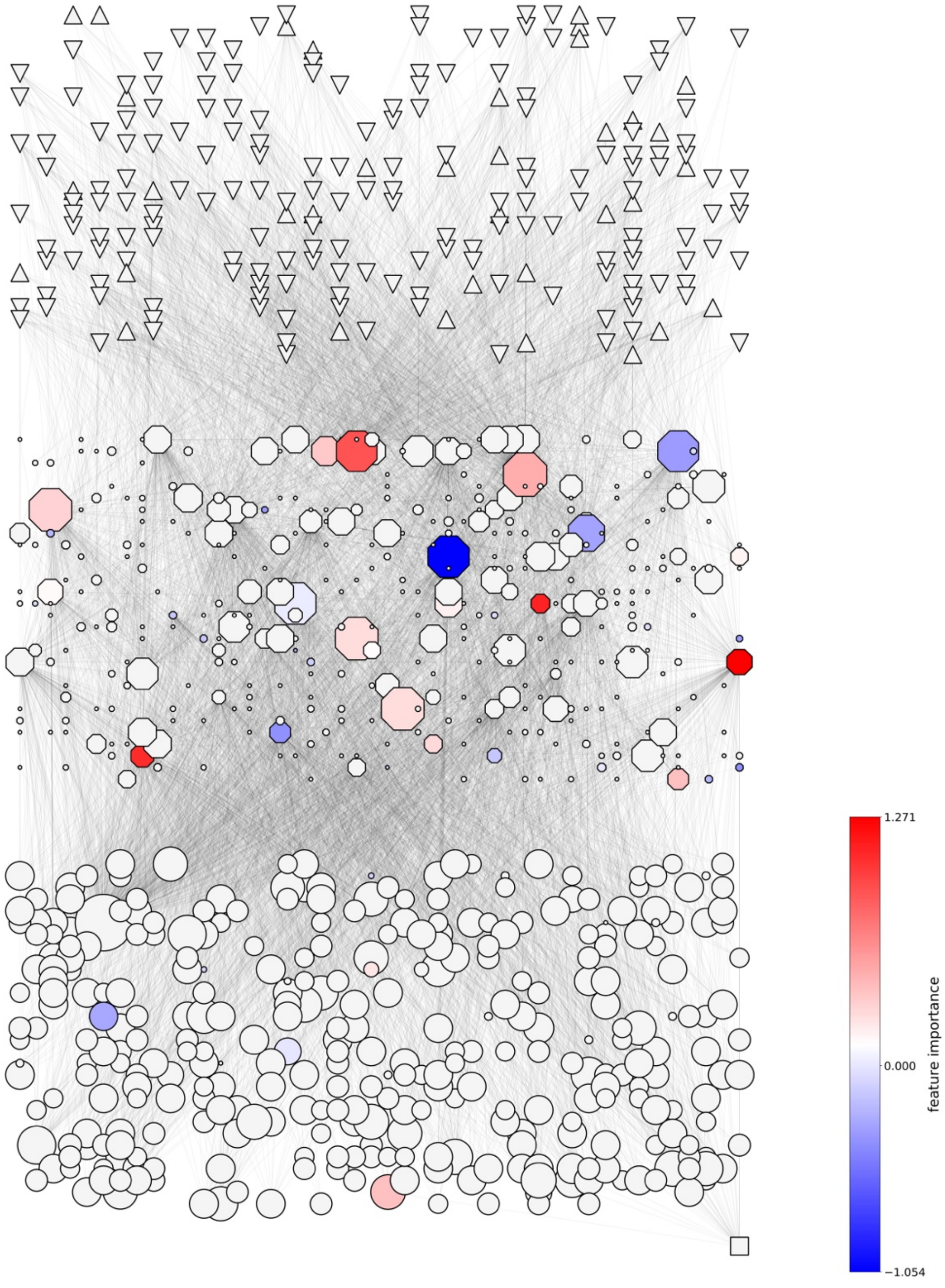

Supplemental Figure 19. Tardive dyskinesia – merged network and feature importance values. The merged interaction network for all true and false positive drugs associated with tardive dyskinesia highlights which network components – drug-binding and network proteins – have high feature importance in the logistic

regression model. True/false positive drugs are represented in the top layer as regular/inverted triangles respectively. Drug-binding and intermediate pathway proteins are represented in the second and third layers. The size of the protein reflects the number of networks in which the protein appears. Relevant tardive dyskinesia-associated phenotypes are represented as boxes in the last layer. Protein coloring reflects the feature importance in the logistic regression model. Red/blue coloring represents association to true/false positive networks.

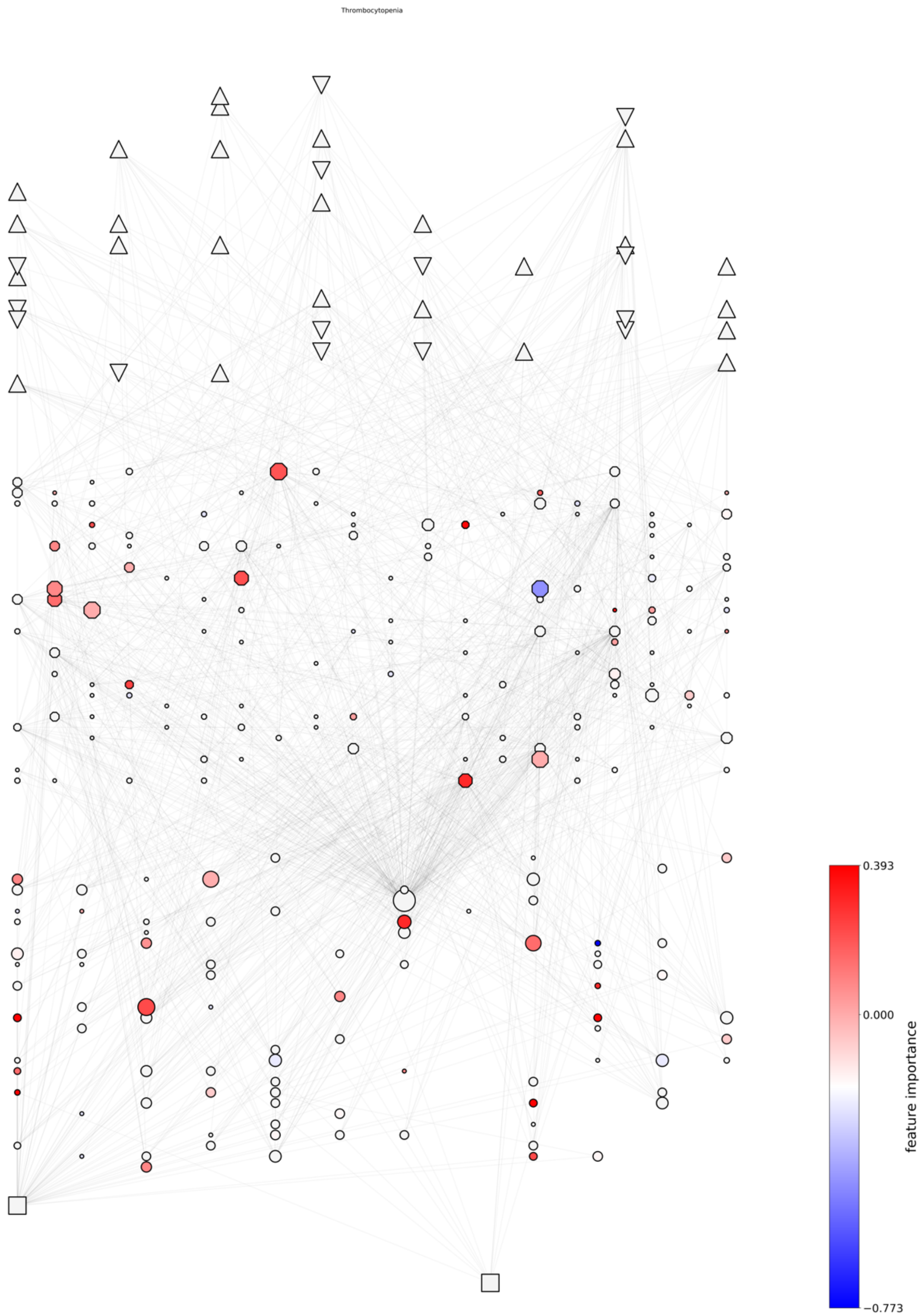

Supplemental Figure 20. Thrombocytopenia – merged network and feature importance values. The merged interaction network for all true and false positive drugs associated with thrombocytopenia highlights which network components – drug-binding and network proteins – have high feature importance in the logistic regression model. True/false positive drugs are represented in the top layer as regular/inverted triangles respectively. Drug-binding and intermediate pathway proteins are represented in the second and third layers.

The size of the protein reflects the number of networks in which the protein appears. Relevant thrombocytopenia-associated phenotypes are represented as boxes in the last layer. Protein coloring reflects the feature importance in the logistic regression model. Red/blue coloring represents association to true/false positive networks.
